# Prior Aerobic Exercise Training Fails to Confer Cardioprotection Under Varying Exercise Volumes in Early Post-Infarction Cardiac Remodeling in Female Rats

**DOI:** 10.3390/biomedicines13092221

**Published:** 2025-09-10

**Authors:** André Rodrigues Lourenço Dias, Ednei Luiz Antonio, Helenita Antonia de Oliveira, Ighor Luiz Azevedo Teixeira, Larissa Emília Seibt, Andrey Jorge Serra

**Affiliations:** Department of Medicine, Cardiology Division, Federal University of São Paulo, Rua Pedro de Toledo, 781, 10° Andar, São Paulo 04039-032, SP, Brazil; andreerld@hotmail.com (A.R.L.D.); elantonio@unifesp.br (E.L.A.); helenitaoli@gmail.com (H.A.d.O.); ighorazevedo@gmail.com (I.L.A.T.); larissa.e.seibt@gmail.com (L.E.S.)

**Keywords:** coronary artery disease, ischemic heart disease, left ventricle, preconditioning

## Abstract

**Background**: There is no information on how the dose of exercise training prior to myocardial infarction (MI) affects cardioprotection. **Objective**: This study aimed to evaluate the cardioprotective role of different volumes of exercise training prior to MI. **Methods**: Wistar female rats were allocated to one of the following groups: SHAM (not trained and undergoing simulated MI surgery), NT+MI (untrained and undergoing MI surgery), T60+MI (trained 60 min per session and undergoing MI surgery), T90+MI (trained 90 min per session and undergoing MI surgery), and T180+MI (trained 180 min per session and undergoing MI surgery). The training protocol was performed in a swimming pool for eight weeks. On the seventh day after MI, the animals underwent left ventricular (LV) structural and functional evaluation and were euthanized for molecular analyses. **Results**: Exercise training groups had greater VO_2_peak and LV mass than did the SHAM group. The MI size did not differ statistically among the experimental groups. Compared with the SHAM group, all the MI groups presented a lower LV shortening fraction. LV systolic pressure was significantly lower in the T60+MI group than in the SHAM and T180+MI groups. The +dP/dt of the LV was significantly lower in the NT+MI, T60+MI, and T90+MI groups than in the SHAM group. We did not find significant changes in the inflammatory mediators and oxidative stress markers as well as proteins involved in calcium handling. **Conclusions**: Exercise training prior to MI enhanced cardiorespiratory fitness and induced LV hypertrophy, however, regardless of volume, was unable to counteract the detrimental effects of MI.

## 1. Introduction

Exercise training prior to myocardial infarction (MI) plays a key role in mitigating the deleterious effects of cardiovascular diseases [1]. Prospective cohort studies have shown that leisure-time physical activity is associated with a 47–60% reduction in the likelihood of fatal myocardial infarction [2,3]. However, these studies have significant limitations concerning the ability to isolate the causal effect of physical activity on the occurrence of fatal MI, making the establishment of a cause–effect relationship challenging.

Experimental studies have shown that exercise training prior to MI has several benefits, such as increased angiogenesis, reduced MI size and LV dysfunction, decreased inflammation, apoptosis, and myocardial fibrosis [1,4]. Under experimental conditions, exercise sessions lasting from 60–90 min are common [4]. However, the ideal training volume to maximize cardioprotection is still unknown. Previous studies have only examined the role of exercise training volume when it is initiated after MI. These studies revealed that a small training volume, 15 min per session, is more effective in inducing cardioprotection than larger training volumes [5,6]. However, it is pivotal to highlight that the heart after MI is in a highly vulnerable state. Therefore, such a small daily training volume may not be ideal for preconditioning a healthy heart against future ischemic insults.

Considering the dose–response curve of physical training in preventing cardiovascular diseases and reducing mortality, it is assumed that, similarly, the volume of training prior to MI does not have a linear relationship with cardioprotection [7,8,9]. In summary, it is assumed that the most relevant benefits of training prior to MI should occur when changing from an inactive lifestyle to moderate training volumes [9,10]. On the other hand, high training volumes may lead to a plateau or even a reduction in cardioprotection [8]. Moreover, extreme training volumes may be detrimental to cardiac health, increasing susceptibility to arrhythmias, fibrosis, and sudden cardiac death [11,12,13,14]. However, there is no information in the literature on how exercise training volume prior to MI affects cardioprotection. Therefore, the present study aimed to evaluate the cardioprotective role of different volumes of exercise training prior to MI.

## 2. Materials and Methods

### 2.1. Sampling

All experimental procedures were performed in accordance with the Guide for the Care and Use of Laboratory Animals, published by the National Institutes of Health (NIH Publication Nº 85-23, revised 1996). The experimental protocol was approved by the Ethics Committee on Animal Use of the Federal University of São Paulo (CEUA/UNIFESP), Brazil (approval number: 9274080819). Female Charles River Wistar IGS rats (9 weeks old) were housed in a facility with temperature control, a 12/12 h light/dark cycle, and access to water and Nuvilab chow ad libitum. Female animals were chosen because of their lower mortality from cardiac ischemia [15,16], which allowed the study to be conducted with a smaller number of subjects. The animals were randomized into the following groups: untrained and underwent simulated MI surgery (SHAM); untrained and underwent MI surgery (NT+MI); trained 60 min per session and underwent MI surgery (T60+MI); trained 90 min per session and underwent MI surgery (T90+MI); trained 180 min per session and underwent MI surgery (T180+MI). The sample size for each dependent variable can be found in the Supplementary Materials (Table S1).

### 2.2. Experimental Design

**Figure 1:**
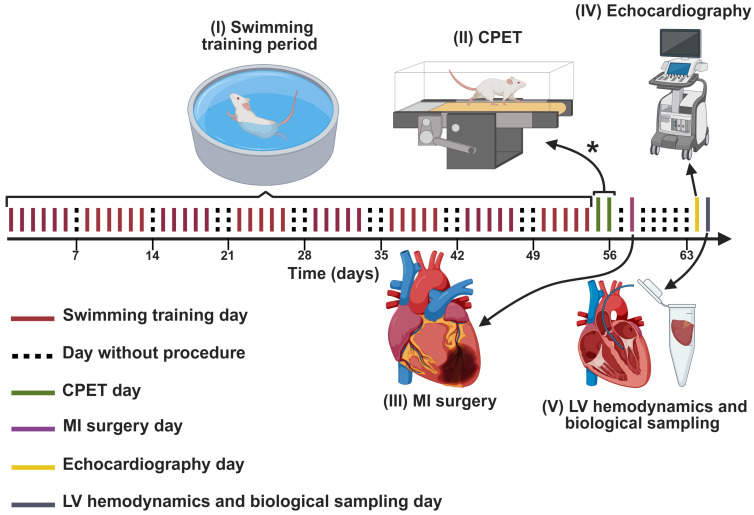
Experimental Design. After the animals were randomized into one of the five groups (SHAM, NT+MI, T60+MI, T90+MI, T180+MI), the following procedures were carried out: (I) the swimming training protocol for eight weeks; (II) one or two days after the last training session, the CPET was performed. Familiarization on the treadmill was conducted during the three days preceding the CPET (not shown in the timeline). * Each animal performed the CPET either on the first or the second day after last swimming training session, but never on both days. (III) four days after the last session training, MI induction surgery was performed; (IV) echocardiographic evaluation was conducted six days after the MI; and (V) LV hemodynamic assessment and biological material collection were performed seven days after MI. CPET: cardiopulmonary exercise test; LV: left ventricle; MI: myocardial infarction.

As illustrated in Figure 1, swimming training was initiated after the rats were allocated to their respective groups. After three days of familiarization on the treadmill, the rats underwent a cardiopulmonary exercise test (CPET) to determine peak oxygen consumption (VO_2_peak), conducted one or two days after the last swimming training session. Four days after the last session training, MI was induced in the NT+MI, T60+MI, T90+MI, and T180+MI groups, while the SHAM group underwent simulated surgery. Echocardiographic examination was carried out six days post-MI, and LV hemodynamic evaluation and biological material collection were performed seven days post-MI. Table S2 in the Supplementary Materials shows which procedures were performed in a blinded manner.

### 2.3. Swimming Training Protocols

The training was carried out in a fiberglass water tank adapted for rodents, with the water temperature maintained at ~32–33 °C. A maximum of 12 rats was placed in the pool together for each session. Training lasted eight weeks and consisted of two phases: (1) In the adaptation phase, the rats swam without any external load for 12 sessions (six sessions per week). The first session lasted 15 min, and 15 min were added to each subsequent session until the target training duration for each group was reached. (2) In the main phase, the rats swam with an external load, starting at 1% of their body mass and increasing every two days, up to a maximum of 4% of body mass. This phase was performed five times per week for six weeks. Training durations of 60 and 90 min per session were chosen because they are traditionally used in experimental swimming studies evaluating the cardioprotective effects of exercise [4]. The 180-min duration was chosen to represent an extreme but tolerable exercise load for rats, analogous to athlete training [17].

### 2.4. CPET

The CPET was performed on a treadmill equipped with a Panlab-Harvard Apparatus gas analyzer (Oxylet System, Harvard Bioscience Company, Holliston, MA, USA) as previously described. The gas analyzer was calibrated before each test using two gas mixtures: (1) 20% O_2_ and 0% CO_2_, and (2) 50% O_2_ and 1.5% CO_2_. The flow rate was maintained at 1 L/min. Prior to each test, for at least two minutes, the ambient air O_2_ and CO_2_ concentrations were recorded and used as estimates of inspired O_2_ and inspired CO_2_, respectively. The following parameters were measured: expired O_2_ and expired CO_2_. VO_2_peak was calculated during the last second of the CPET. All the animals were familiarized for three consecutive days at a progressive intensity for 15 min. When treadmill familiarization was performed on the same day as the swimming training session, familiarization was always conducted first, and a minimum interval of one hour was ensured between the treadmill familiarization and the training session. The CPET was conducted one day after the last treadmill adaptation session and one or two days after the last swimming training session. The CPET protocol was progressive, starting at 25 cm/s without inclination. The treadmill speed was increased by 9 cm/s every two minutes until the animal was unable to run despite exposure to an electrical stimulus for four seconds. The electrical stimulus was gradually increased up to 2 mA, as needed, to keep the animal active.

### 2.5. MI Model

MI was induced by permanent left anterior descending coronary occlusion following anesthesia with a mixture of ketamine (80 mg/kg) and xylazine (10 mg/kg) (i.p.). The rats were intubated and ventilated at a rate of 90–100 breaths per minute, with a tidal volume of 10 mL/kg and positive pressure using a rodent ventilator, model 7025 Ugo Basile (Biological Research Apparatus, Comerio, Italy). After dissection of the intercostal muscle, the ribs were retracted using a Kelly clamp and a hinged Lasik retractor with a plate adapted for the thorax (DL Micof, São Paulo, Brazil). After the heart was exteriorized, the coronary artery was ligated approximately 5 mm from its origin at the aorta using a 5.0 nylon suture with a circular needle. The thorax was closed with a purse-string suture previously prepared around the incision edges. The rats were then maintained on artificial ventilation enriched with O_2_ until spontaneous breathing movements occurred. The SHAM group underwent the same surgical procedures but received suture insertion without coronary occlusion.

### 2.6. Echocardiography

Echocardiographic examination was performed using an HP SONOS 5500 system (Hewlett-Packard, Andover, MA, USA) equipped with a 12 MHz transducer and a depth setting of 3 cm. The rats were anesthetized with ketamine (80 mg/kg) plus xylazine (10 mg/kg) (i.p.). After shaving the anterior thoracic region, the rats were positioned in the left lateral decubitus position, and ethyl alcohol (70%) was applied to the thoracic region. The transducer was then covered with a gel specifically for ultrasound examination. B-mode images were acquired in the right parasternal short-axis view. Pulsed Doppler measurements were obtained in the apical four-chamber view. Images were stored on VHS tape (Nipponic, EHG T160, Manaus, Brazil). MI size was determined by identifying akinetic regions of the LV wall [18]. There is strong agreement between MI size assessment by echocardiography and histology when evaluated 7 days after MI [18]. At the end of LV diastole, MI size was measured as the sum of the infarct lengths in the apical and papillary regions of the LV relative to the sum of the LV circumference of these two regions:

$$MI\;size\;(\%)=\frac{\left(MI\;length\;in\;papillary\;region+MI\;length\;in\;apical\;region\right)}{\left(LV\;circumference\;in\;papillary\;region+LV\;circumference\;in\;apical\;region\right)}\times 100$$

### 2.7. LV Hemodynamics

Seven days after MI, the animals were anesthetized with urethane (1.6 g/mL/kg, i.p., in bolus) (Sigma, St. Louis, MO, USA), positioned in dorsal decubitus, and kept warm ~37 °C using a heating pad (Homeothermic blanket system, Harvard Apparatus, model 507053F, Edenbridge, UK). A Millar micromanometer (MikroTip^®^ 2F, Millar Instruments Inc., Houston, TX, USA) was inserted in the LV cavity via right carotid catheterization. To stabilize LV diastolic pressure, 0.5–1 mL of hydroxyethyl starch (Voluven^®^) was slowly infused through the femoral vein. Data were collected 5–10 min after hydroxyethyl starch administration and recorded via the LabChart PowerLab 4/30 acquisition system (ADInstruments PowerLab^®^, ADInstruments Ltd., Bella Vista, Australia). The instantaneous parameters obtained included heart rate (HR), LV systolic pressure (LVSP), LV end-diastolic pressure (LVEDP), the lowest derivative value of the LV pressure curve (−dP/dt), the highest derivative value of the LV pressure curve (+dP/dt), diastolic blood pressure (DBP), and systolic blood pressure (SBP).

### 2.8. Euthanasia and Collection of Biological Materials

The animals were euthanized with an overdose of urethane (1.8 g/mL/kg; i.p.; in bolus) (Sigma). Total ventricular mass was measured after dissection of the atriums and great vessels at the base of the heart. Following removal of the right ventricle (RV), the LV mass (LVM) was determined. The RV mass (RVM) was calculated as the difference between total ventricular mass and LVM. Both LVM and RVM were normalized to body mass and expressed as mg/g of body mass. LV samples separated for analysis belonged to remote area, corresponding to the interventricular septum/posterior free wall of the LV. Samples were stored in an ultrafreezer at −80 °C until further processing. The right lung was isolated by tying a cotton thread around the pulmonary hilum to prevent fluid loss during handling. The lungs were removed and immediately weighed to determine wet mass. Tissue samples were kept in an oven (90 °C for 48 h), after which the dry mass of the lungs was obtained. Lung water content (LWC) was calculated using the following formula: LWC = [(wet mass − dry mass)/wet mass] × 100. The soleus muscle mass (SMM) was obtained from the right hind limb.

### 2.9. ELISA Assays

Approximately 65 mg of LV tissue was homogenized in lysis buffer (50 mM Tris-HCl, 0.5% SDS, and 1 mM DTT, pH 8.0) containing protease inhibitors diluted 1:300 (Sigma Aldrich, St. Louis, MO, USA). The homogenate was centrifuged (Centrifuge 5415 R, Eppendorf, Hamburg, Germany) at 1500× *g* for 10 min at 4 °C, and the resulting supernatant was collected for the determination of interleukin-6 (IL-6), interleukin-1-beta (IL-1β), interleukin-10 (IL-10), tumor necrosis factor-alpha (TNF-α), and vascular endothelial growth factor (VEGF). EIA/RIA microplates (R&D Systems, Minneapolis, MN, USA) were coated for 14–16 h at room temperature with 100 μL/well of capture antibody solution diluted in PBS (137 mM NaCl, 2.7 mM KCl, 8.1 mM Na_2_HPO_4_; 1.5 mM KH_2_PO_4_, pH 7.2–7.4, 0.2 μm filtered). The microplates were then incubated with 100 μL/well of biotinylated detection antibody solution diluted in PBS containing 1% BSA. After washing, the wells were incubated with 100 μL of streptavidin-horseradish peroxidase solution diluted in PBS with 1% BSA. Absorbances of the standard curves and samples were measured at 450 nm using a spectrophotometer (SpectraMax M5, Molecular Devices, San Jose, CA, USA). The absorbance values were normalized to the total protein content determined by the Bradford method.

### 2.10. Antioxidant Enzyme Activity

For glutathione peroxidase activity (GPX), 180 µL of reaction medium consisting of 50 mL of 50 mM Tris-HCl, 1 mM EDTA, 0.2 mM NADPH, 0.1 U/mL glutathione reductase (G3664 SIGMA), and 1 mM reduced glutathione, pH 7.6, was added to each well of a microplate containing 10 µL of centrifuged homogenate. The plate was subsequently placed in a spectrophotometer at 25 °C for 1–2 min. A total of 10 µL of 0.5 mM TBOOH was added to each well using a multichannel pipette. Absorbance was recorded at 340 nm with a Spectra MaxM5 spectrophotometer (Molecular Devices, San Jose, CA, USA) for 5 min, at 30-s intervals, at 25 °C. GPX activity was normalized to total protein concentration measured by the Bradford method and expressed as enzyme units per milligram of total protein (U/mg protein).

The superoxide dismutase (SOD) activity was assessed in a 96-well microplate preheated to 25 °C. Each well contained 25 μL of sample, 200 μL of reaction medium (0.0015 g of blue tetrazolium chloride and 0.0042 g of βNADH in 15 mL of phosphate buffer [50 mM, 0.1 mM EDTA], and 25 μL of phenazine methosulfate [0.0010 g in 50 mL of deionized water]). Eleven absorbance readings were recorded over 5 min at 560 nm using a spectrophotometer.

For catalase (CAT) activity, 20 μL/well of standard, blank, or diluted sample was pipetted followed by the addition of 100 μL of 100 mM phosphate buffer (pH 7.0) 30 μL of 100% methanol, and 20 μL of 35.28 mM H_2_O_2_ (prepared on the day of use). After 20 min, 30 μL of 10 M potassium hydroxide was added to stop the reaction. Subsequently, 30 μL of 72.83 mM purpald was added, and the mixture was incubated with agitation for 10 min. Then, 10 μL of 190.8 mM potassium periodate was added, and the mixture was incubated for 5 min. The microplate was further incubated in an oven for 5–10 min, after which absorbance was measured at 540 nm for 20 min. CAT activity was normalized to total protein concentration determined by the Bradford method and expressed as nmol/min/mg of total protein.

### 2.11. Western Blotting

Total protein quantification was performed via the Bradford method. Approximately 15–30 μg/μL protein was subjected to SDS–polyacrylamide gel electrophoresis (10–12%, Bio-Rad gel, USA). After separation, the proteins were transferred to nitrocellulose membranes (Hybond-P, AmerSham Biosciences; Piscataway, NJ, USA). The membranes were blocked for 1 h in TBST (Tris-base 50 mM; NaCl 1.5 M; Tween 20 0.1%; pH 7.4) containing skim milk (5%). The transferred proteins were then incubated at ~4 °C (overnight) with the following primary rabbit antibodies: anti-L-type high-voltage calcium channel Ca_v_1.2 (LTCC) (1:10,000 ACC-003, Alomone, Jerusalem, Israel), anti-sarco/endoplasmic reticulum Ca_2+_-ATPase (SERCA2a) (1:1000 A010-20, Badrilla, Leeds, UK), anti-phospholamban phosphorylated at threonine 17 and serine 16 (PLB(Thr17/Ser16)) (1:5000 ab62170, Abcam, Cambridge, MA, USA), anti-sodium/calcium exchanger 1 (NCX1) (1:5000 ab177952, Abcam, Cambridge, MA, USA), anti-4-hydroxynonenal (4-HNE) (1:4000 ab46545, Abcam, Cambridge, MA, USA), and anti-GAPDH (1:20,000 D16H11, Cell Signaling Technology, Danvers, MA, USA), as well as the following primary mouse antibodies: anti-ryanodine receptor (RyR) (1:2000 ab2868, Abcam, Cambridge, MA, USA), and anti-total phospholamban. All investigated proteins were normalized to the GAPDH content. The membranes were then washed five times (5 min/wash) in TBST buffer and incubated for 60 min at room temperature with secondary anti-rabbit or anti-mouse antibodies (diluted 1:2000). Following incubation, the membranes were washed again with TBST (five times; 5 min each wash) and exposed to enhanced chemiluminescence (ECL) reagent (Bio-Rad, Hercules, CA, USA). Molecular weight markers (Bio-Rad) were used to identify the proteins. Protein expression was quantified after scanning the membranes using a gel documentation system (AmerSham Images 600, GE HealthCare, Chicago, IL, USA). For image analysis, the minimum profile was used as the background method, a 4% median filter was applied for peak detection, and the edge parameters were automatically detected by ImageQuant TL 1D Software (v8.2.0, General Electric Company, Cincinnati, OH, USA). The data were normalized by protein volume (micrograms).

### 2.12. Statistical Analysis

Assuming a large effect size = 0.4, α = 0.05, and power = 0.8, a sample size of 16 animals per group is required for the F-test in a one-way ANOVA with independent measures. Analyses were performed using R software (Version 4.4.0). Pearson’s chi-square test was applied to assess the association between post-MI mortality and training volume. A proportion test stratified by group or outcome (survived or died) was conducted, and *p*-values were displayed in bar charts for categorical variables using the ggstatsplot package (Version 0.13.1) [19]. Kolmogorov–Smirnov and Levene’s tests were applied to test the normality and homogeneity of the data, respectively. When both assumptions were met, Fisher’s one-way ANOVA was applied, and pairwise comparisons were performed using Student’s *t* test. When normality was met but homogeneity was violated, Welch’s one-way ANOVA was used, with pairwise comparisons conducted via the Games–Howell test. When the data did not exhibit normality, heteroscedastic one-way ANOVA (Welch’s ANOVA with trimmed mean) [20] was used, and pairwise comparisons were performed via Yuen’s trimmed test. Means (when normality was present) or 20% trimmed means (when normality was absent) were chosen as measures of central tendency. The 95% confidence interval (CI 95%) of the mean or trimmed mean was calculated using the Tukey–McLaughlin method with the trimci function from the WRS2 package (Version 1.1-7). *p*-value adjustment for multiple comparisons was performed using the Holm test (default post hoc method in the ggstatsplot package). A significance level of *p* < 0.05 was adopted.

## 3. Results

### 3.1. Post-MI Mortality

There were no significant differences in mortality seven days after MI (NT+MI: 12%; T60+MI: 31%; T90+MI: 25%; T180+MI: 20%) (Supplementary Materials, Figure S1(A1)). When only the rats that died post-MI were analyzed, the proportions of animals in each group did not differ significantly (*p* = 0.3) (Supplementary Materials, Figure S1(A2)).

### 3.2. Biometric and CPET Data

**Figure 2:**
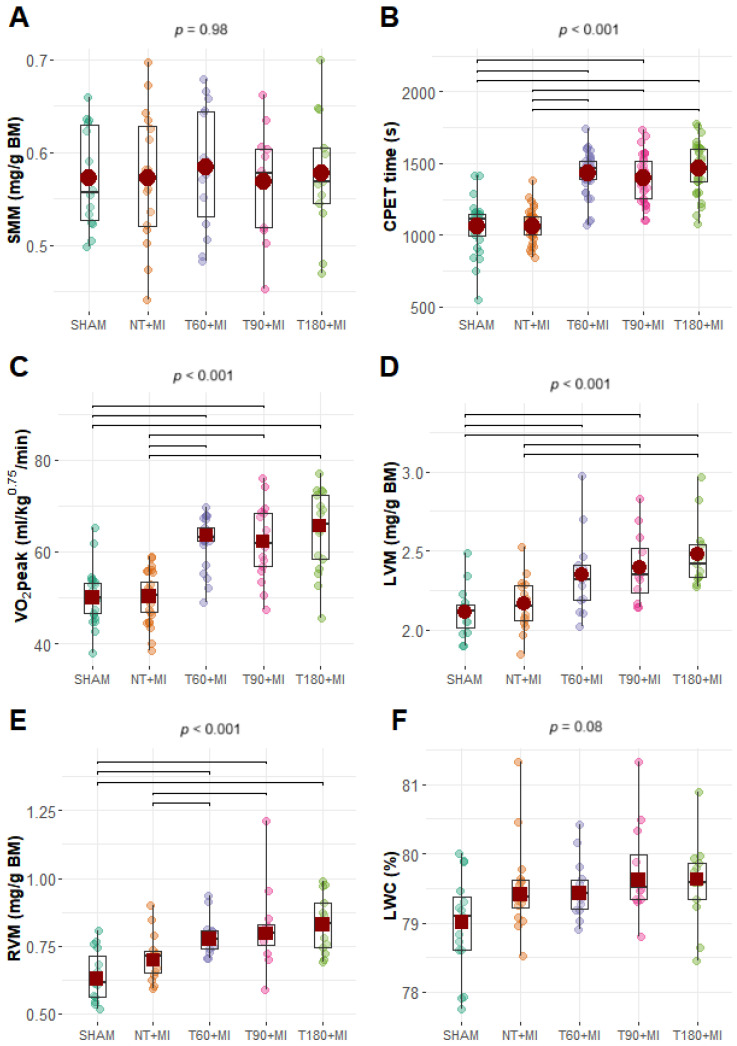
Biometric and CPET data. (**A**) SMM, soleus muscle mass; (**B**) CPET time, cardiopulmonary exercise test time; (**C**) VO_2_peak, peak oxygen consumption; (**D**) LVM, left ventricular mass; (**E**) RVM: right ventricular mass; (**F**) LWC, lung water content. BM: body mass. Boxplots (black squares with whiskers) with individual data points (small circles of different colors) and means (red circles) or 20% trimmed means (red squares). The *p*-value displayed above the boxplots refers to the ANOVA. Brackets were added to the graphs to represent significant differences in pairwise comparisons. (**A**,**B**,**D**) charts: Fisher’s one-way ANOVA and pairwise comparisons via Student’s *t* test. (**C**,**E**,**F**) charts: Welch’s ANOVA with trimmed means and pairwise comparisons via Yuen’s trimmed test.

The body mass of the rats increased over the weeks (Supplementary Materials, Figure S2A); however, there were no differences between the groups after the swimming training ended (Figure S2B) and six days after MI (Figure S2C). Compared with the SHAM and NT+MI groups, all the trained groups presented greater VO_2_peak and CPET times (Figure 2B,C). There were no significant differences between the groups in terms of SMM or LWC (Figure 2A,F). LVM was greater in the T180+MI and T90+MI groups than in the SHAM and NT+MI groups, and the T60+MI group presented greater LVM than the SHAM group (Figure 2D). RVM was greater in the T60+MI and T90+MI groups than in the SHAM and NT+MI groups, and the T180+MI group had a greater RVM than the SHAM group (Figure 2E).

### 3.3. Echocardiographic and Hemodynamic Data

**Figure 3:**
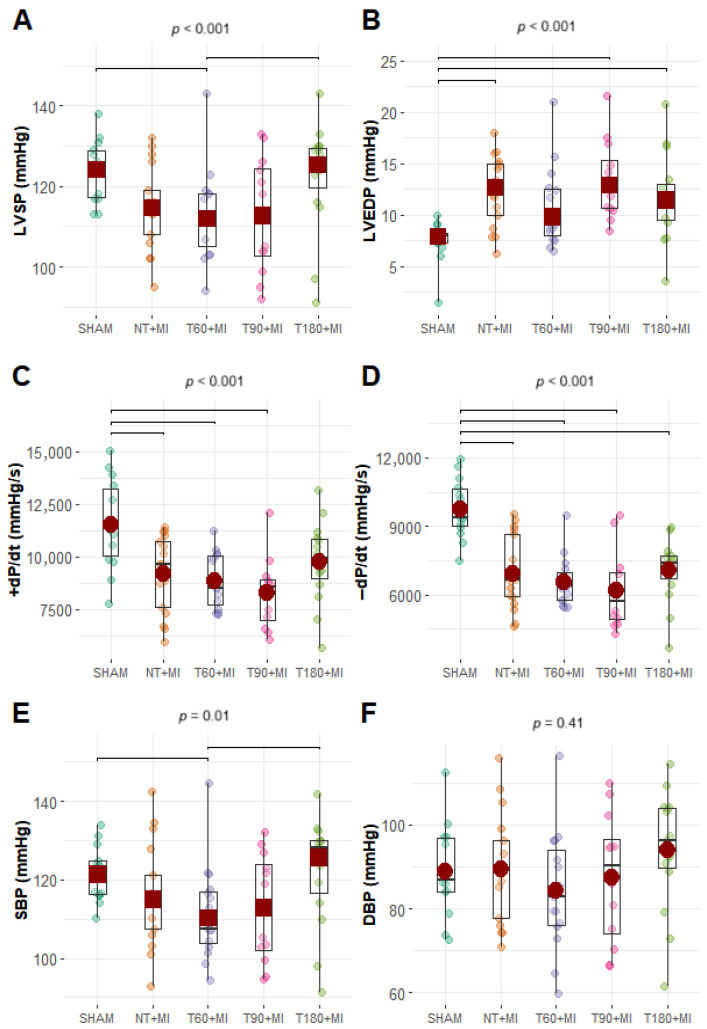
Parameters of the intraventricular and aortic pressure curves during the cardiac cycle. (**A**) LVSP, left ventricular end-systolic pressure; (**B**) LVEDP, left ventricular end-diastolic pressure; (**C**) +dP/dt, maximum value of the derivative of the left ventricular pressure curve; (**D**) −dP/dt, minimum value of the derivative of the left ventricular pressure curve; (**E**) SBP, systolic blood pressure; (**F**) DBP, diastolic blood pressure. Boxplots (black squares with whiskers) with individual data points (small circles of different colors) and means (red circles) or 20% trimmed means (red squares). The *p*-value displayed above the boxplots refers to the ANOVA. Brackets were added to the graphs to represent significant differences in pairwise comparisons. (**C**,**D**,**F**) charts: Fisher’s one-way ANOVA and pairwise comparisons via Student’s *t* test. (**A**,**B**,**E**) charts: Welch’s ANOVA with trimmed means and pairwise comparisons via Yuen’s trimmed test.

**Figure 4:**
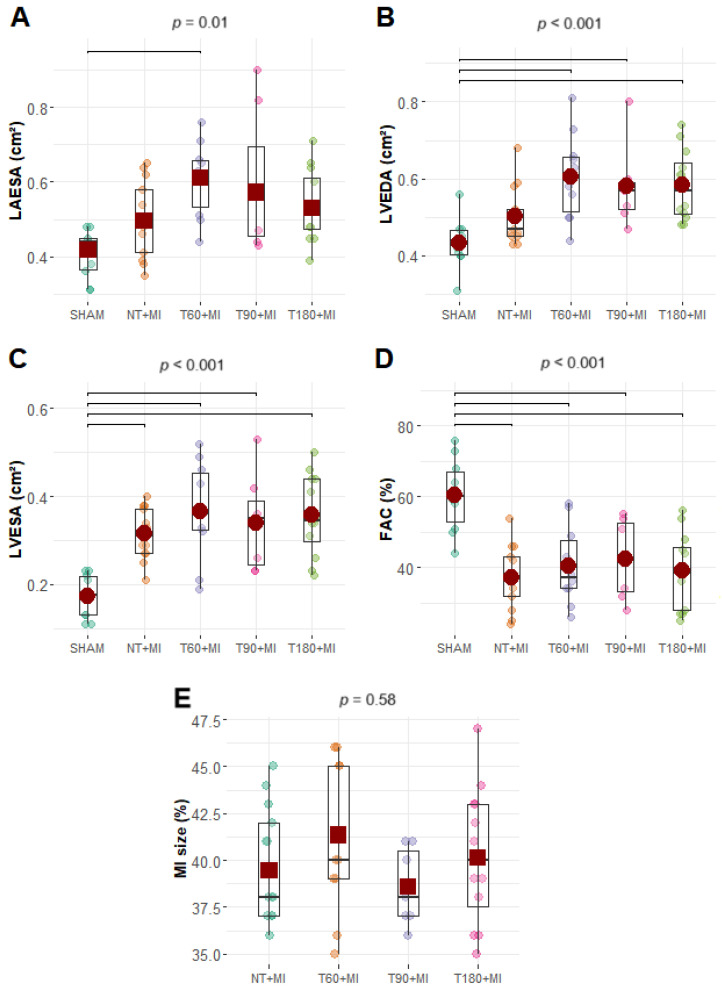
Echocardiographic parameters of cardiac remodeling. (**A**) LAESA, left atrial area at end-systole; (**B**) LVEDA, left ventricular end-diastolic area at the papillary muscle level; (**C**) LVESA, left ventricular end-systolic area at the papillary muscle level; (**D**) FAC, fractional area change in the left ventricular cross-sectional area; (**E**) MI size, myocardial infarction size. Boxplots (black squares with whiskers) with individual data points (small circles of different colors) and means (red circles) or 20% trimmed means (red squares). The *p*-value displayed above the boxplots refers to the ANOVA. Brackets were added to the graphs to represent significant differences in pairwise comparisons. (**B**–**D**) charts: Fisher’s one-way ANOVA and pairwise comparisons via Student’s *t* test. (**A**,**E**) charts: Welch’s ANOVA with trimmed means and pairwise comparisons via Yuen’s trimmed test.

The HR (F_Fisher_, *p* = 0.69) and DBP did not differ significantly among the experimental groups (Supplementary Materials, Figure S3), but the LVSP and SBP were significantly lower in the T60+MI group than in the SHAM and T180+MI groups (Figure 3A,E). LVEDP was significantly lower in the SHAM group than in the NT+MI, T90+MI, and T180+MI groups but did not differ significantly from that in the T60+MI group (Figure 3B). The +dP/dt was significantly greater in the SHAM group than in the NT+MI, T60+MI, and T90+MI groups, and there was no difference compared with the T180+MI group (Figure 3C). Compared with the SHAM group, all MI groups presented lower -dP/dt values (Figure 3D) (expressed in positive values). Representative images from the echocardiographic assessment can be seen in Figure S4. On echocardiographic examination (Figure 4E), MI size did not differ statistically among the experimental groups. The T60+MI group exhibited a larger left atrial area at end-systole (LAESA) compared with the SHAM group (Figure 4A). Compared with the SHAM group, all trained groups presented a greater LV end-diastolic area (LVEDA) (Figure 4B). Compared with the SHAM group, all MI groups had a greater LV end-systolic area (LVESA) and a lower fraction of shortening of the LV cross-sectional area (FAC) (Figure 4C,D). Diastolic echocardiographic parameters did not differ statistically between the groups (Table 1).

### 3.4. Cytokines, Oxidative Stress Antioxidant Enzymes, and Endothelial Markers

The levels of cytokines (IL-6, IL-10, IL-1β, and TNF-α), oxidative stress marker (4-HNE), antioxidant enzymes (CAT, SOD, GPX), and VEGF did not differ among the experimental groups (Table 2).

### 3.5. Calcium-Handling Proteins

**Figure 5:**
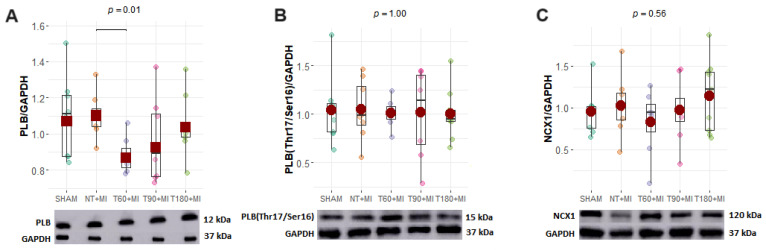

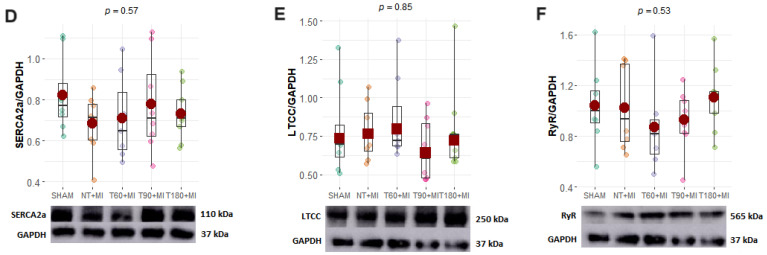
Calcium-handling proteins. (**A**) PLB, total phospholamban; (**B**) PLB(Thr17/Ser16), phospholamban phosphorylated at serine 16 and threonine 17; (**C**) NCX1, sodium/calcium exchanger 1; (**D**) SERCA2a, sarco/endoplasmic reticulum Ca^2+^-ATPase; (**E**) LTCC, L-type Ca_v_1.2 voltage-gated calcium channel; (**F**) RyR, ryanodine receptor. GAPDH: glyceraldehyde 3-phosphate dehydrogenase constitutive protein. Boxplots (black squares with whiskers) with individual data points (small circles of different colors) and means (red circles) or 20% trimmed means (red squares). The *p*-value displayed above the boxplots refers to the ANOVA. Brackets were added to the graphs to represent significant differences in pairwise comparisons. (**B**–**D**,**F**) charts: Fisher’s one-way ANOVA and pairwise comparisons via Student’s *t* test. (**A**,**E**) charts: Welch’s ANOVA with trimmed means and pairwise comparisons via Yuen’s trimmed test. The GAPDH-LTCC and GAPDH-RyR are equivalent because the same membrane was used to detect LTCC and RyR through membrane stripping for Western blotting.

Most calcium-handling proteins did not differ significantly among the experimental groups (Figure 5). An exception was PLB, which was lower in the T60+MI group than in the NT+MI group (Figure 5A).

## 4. Discussion

The present study demonstrates that, regardless of the volume of aerobic exercise training prior to MI, there was no evidence of cardioprotection. None of the exercise volumes tested (60, 90, or 180 min per session) were effective in reducing MI size or improving left ventricular functional parameters following MI.

All exercise training protocols were effective in improving cardiorespiratory fitness (Figure 2C), indicating a plateau trend in VO_2_peak with a swimming volume of 60 min per session, in line with previous studies [21,22,23]. Although the SHAM and NT+MI groups did not undergo structured training, they were nonetheless exposed to short-term exercise effects through CPET performed 48–72 h before MI induction. In the present study, mortality was 12% in the NT+MI group and 31% in the T60+MI group. In contrast, a previous investigation from our laboratory, which also employed female Wistar rats but without prior CPET, reported 36% mortality (18/50) in untrained rats within the first 24 h following permanent coronary occlusion [24]. Importantly, as little as two exercise sessions have been shown to confer cardioprotection against ischemia–reperfusion for up to 60 h after the last session, significantly reducing MI size and the incidence of ventricular fibrillation [25]. More recently, Ma et al. [26] demonstrated that a single 60-min session of moderate-intensity treadmill exercise, performed 72 h prior to the induction of calcium overload in an in vitro cardiac model, significantly reduced MI size. Taken together, these findings suggest that the relatively low mortality observed in the NT+MI group may have resulted from cardioprotective effects elicited by the CPET protocol.

On echocardiographic evaluation, there was no effect of exercise training on MI size (Figure 4E). However, studies involving exercise training prior to MI have reported smaller MI through histopathological analyses [27,28,29] but not when using echocardiography [24,30,31,32]. Therefore, it is not possible to establish whether exercise training does not reduce MI size or if echocardiography lacks sufficient sensitivity to assess MI size under these conditions.

Cardiac repair after MI can be divided into three phases: the acute inflammatory phase (up to 4 days), the resolution and healing phase (4–14 days), and the cardiac remodeling phase (>14 days) [33]. In an ischemia–reperfusion model, there is initially an increase in the cytokines IL-6 and IL-1β, but by the seventh day after ischemia, these cytokine levels usually normalize [34]. In contrast, IL-10 and TNF-α levels in the LV do not increase during the first seven days after ischemia–reperfusion injury [34]. Data obtained six days after MI show normal expression of IL-6 and IL-1β mRNAs in the infarcted and remote areas in rats [35], which may be explained by lower expression of these mRNAs by macrophages localized in the LV [34]. The present study targeted seven days post-MI, a period when acute inflammation has already subsided, which explains the absence of changes in cytokine levels (IL-6, IL-10, IL-1β, and TNF-α) in the LV (Table 2). We also did not identify alterations in antioxidant enzymes (SOD, CAT, and GPX) post-MI (Table 2), corroborating the findings of Hill and Singal [36]. In contrast, seven days after ischemia–reperfusion, a reduction in antioxidant enzyme activity has been reported [37], suggesting that the antioxidant response induced by permanent occlusion differs from that induced by ischemia–reperfusion.

There was no increase in the LVEDA (Figure 4B), LVM (Figure 2D), and RVM (Figure 2E) in the NT+MI group seven days after MI. Nevertheless, other studies have reported an increase in the LV diastolic diameter one week after MI, and exercise training prior to MI does not always prevent such ventricular remodeling [28,30]. In the present study, the three trained groups presented greater LVEDA (Figure 4B), LVM (Figure 2D), and RVM (Figure 2E) values than the SHAM group, indicating that exercise training induces cardiac hypertrophy and remodeling, regardless of the training volume. A recently published meta-analysis demonstrated that in men, but not in women, there is a positive relationship between training duration and LV hypertrophy [38]. Conversely, experimental studies involving swimming training in female rats revealed that training volumes greater than 60 min per day promote greater LV hypertrophy [21,23]. In addition, in these studies conducted in healthy animals, training volume was increased by raising the number of daily sessions [21,23]. Evidence is lacking on how training programs with the same weekly volume, but different numbers of daily sessions influence LV hypertrophy.

Systolic LV function is directly impacted by MI, resulting in a reduction in FAC, which is not minimized by prior exercise training, whether evaluated between 1 and 28 days after MI [24,30,39,40]. To generate smaller MI, coronary artery occlusion was performed 5 mm from its origin in the aorta instead of 3 mm, as in other studies [24,30]. Nevertheless, exercise training prior to MI did not confer any benefit on FAC (Figure 4D), regardless of the training volume. The greater LVSP observed in the T180+MI group compared with the T60+MI group (Figure 3A) indicates that, similar to exercise intensity [28], the volume of exercise training prior to MI is also important to increase LVSP. Calcium-handling proteins do not appear to explain how training volume influences LVSP. The expression of NCX1 (Figure 5C), the main calcium-handling protein involved in the Anrep effect [40], did not differ between the groups with different training volumes. In the absence of substantial changes in the expression of calcium-handling proteins, the increase in LVM induced by training preconditioning was not sufficient to confer benefits on either diastolic or systolic cardiac function. Our findings reinforce the notion that, rather than the mere amount of contractile mass, cardiac function is more closely determined by the kinetics of intracellular calcium.

In our study, the different exercise training protocols did not attenuate diastolic dysfunction caused by MI, as suggested by the lower values of −dP/dt (Figure 3D). These data do not corroborate previous findings [24,32]. For example, Bozi et al. [29] reported attenuation of damage to −dP/dt with treadmill exercise training prior to MI, which was associated with a lower level of cardiac fibrosis. Although diastolic dysfunction was observed in our study (Figure 3B,D), there was insufficient time for the infarcted animals to develop pulmonary congestion, as evidenced by normal LWC levels (Figure 2F). The main mechanisms that may contribute to LV diastolic dysfunction include fibrosis formation and post-translational modifications of titin or microtubules, which result in increased myocardial stiffness [41,42].

Although exercise training generally does not alter the PLB levels [43,44], a study conducted by Morissette et al. [45] demonstrated that five months of voluntary running reduced PLB content in mice. In our study, a reduction in PLB was found only in the T60+MI group (Figure 5A). Under conditions of increased RyR phosphorylation, PLB ablation intensifies sparks and Ca^2+^ leakage from the sarcoplasmic reticulum, contributing to more pronounced mitochondrial and cytoplasmic Ca^2+^ overload [46,47,48]. During ischemia, increased RyR phosphorylation at serine 2814 leads to greater cytoplasmic and mitochondrial Ca^2+^ overload, thereby increasing the risk of death, especially during the first hour of ischemia [49]. Gazdag et al. [13] demonstrated that a training volume of 200 min/session led to a greater incidence of extrasystole and spontaneous Ca^2+^ release events from the sarcoplasmic reticulum in Wistar rats than in sedentary rats in an induced hypokalemia model. However, the study by Gazdag et al. [13] does not allow for inferences about how smaller training volumes affect the frequency of spontaneous Ca^2+^ release events from the sarcoplasmic reticulum.

Finally, our findings contrast with previous studies in male rats, in which prior exercise training reduced MI size and improved cardiac function [27,50]. This discrepancy may be attributed to the sex of the animals, supporting the hypothesis that sex differences can significantly influence the cardiac response to exercise and ischemic injury. Paroo et al. [51] showed that exercise performed after MI improved cardiac function and reduced LVEDP in males, but did not produce beneficial effects in females. Similarly, Thorp et al. [52] reported that treadmill training increased myocardial tolerance to ischemia/reperfusion in male rats, whereas female rats did not exhibit this benefit. The reasons for these differences are not yet fully understood, but one possibility is that pathological mechanisms in males—unlike those in females—are less influenced by hormonal fluctuations such as those occurring during the estrous cycle. Therefore, the molecular mechanisms underlying the discrepancies between the results of the present study in females and those reported in males still require further investigation.

Although exercise training prior to MI did not attenuate the cardiac damage induced by permanent coronary occlusion, the potential role of exercise in cardiac prehabilitation for patients at risk of MI, including women, should not be underestimated. Robust evidence indicates that exercise contributes to the prevention and mitigation of coronary occlusion by reducing atheromatous plaque volume [53], slowing the progression of stenosis [54], and enhancing blood flow in partially obstructed vessels [55]. Many of the cardiac benefits conferred by exercise training are rapidly lost during detraining [56], including training-induced cardiac hypertrophy [57,58]. Consequently, it appears unlikely that exercise-induced cardiac preconditioning would provide benefits during the late phase of cardiac remodeling that are not already evident within the first week following MI.

### Limitations

Some study limitations should be noted: (i) capillary density and MI size were not directly measured histologically, which could have provided further insights into the implications of our molecular findings; (ii) MI size was not evaluated in the basal region of the LV, making it challenging to compare our results with those of previous studies; (iii) due to logistical issues, hemodynamics were assessed only under basal conditions. Evaluating hemodynamics during sudden afterload increases, with LV pressure and flow measurements, could have provided valuable information about cardiac reserve.

## 5. Conclusions

All three exercise training volumes similarly increased cardiorespiratory fitness and induced LV hypertrophy and remodeling. Exercise training prior to MI, regardless of volume, was unable to counteract the detrimental effects of MI. Future studies should investigate how training frequency, when matched for total weekly volume, as well as sex-related differences, influences cardioprotection.

## Figures and Tables

**Table 1:** Diastolic echocardiographic parameters.

	SHAM	NT+MI	T60+MI	T90+MI	T180+MI	*p*
E-wave (m/s)	0.86 (0.77–0.94)	0.74 (0.68–0.79)	0.84 (0.76–0.93)	0.86 (0.74–0.98)	0.81 (0.73–0.89)	0.10
A-wave (m/s)	0.23 (0.19–0.27)	0.20 (0.16–0.25)	0.19 (0.17–0.21)	0.26 (0.14–0.39)	0.24 (0.17–0.31)	0.15
E/A (a.u.)	3.78 (3.2–4.37)	3.93 (3.16–4.69)	4.56 (4.11–5.02)	3.56 (2.24–4.88)	4.21 (2.81–5.60)	0.17
DT (ms)	42.7 [25.8–59.5]	38.9 [32–45.7]	36.7 [29.1–44.2]	39.4 [27–51.8]	40.7 [37.9–43.6]	0.79
IVRT (ms)	23.2 [19.3–27.1]	24 [19.7–28.3]	25.7 [19.9–31.5]	25.2 [19.3–31.1]	23.6 [18.5–28.8]	0.90

A-wave: peak velocity of the mitral flow during the atrial contraction phase; DT: deceleration time of the E-wave; E/A: ratio between the E-wave and A-wave; E-wave: peak velocity of the mitral flow during the rapid filling phase; IVRT: isovolumetric relaxation time; *p*: *p*-value ANOVA. The data between parentheses and brackets represent the 95% confidence intervals for the mean and trimmed mean, respectively.

**Table 2:** Cytokines, oxidative stress markers, antioxidant enzymes, and angiogenesis markers.

	SHAM	NT+MI	T60+MI	T90+MI	T180+MI	*p*
IL-10 (pg/mg protein)	34.9 [29.7–40]	28.4 [25.8–31.1]	29.3 [25.5–33]	32.3 [26.2–38.4]	29.6 [24–35.2]	0.12
IL-6 (pg/mg protein)	70.4 (64.1–76.7)	70.8 (64.6–76.9)	71.6 (67–76.3)	73.5 (66.7–80.3)	71.4 (64.6–78.3)	0.96
IL-1β (pg/mg protein)	12.6 (9.4–15.7)	11.2 (8.5–13.8)	13.8 (11–16.6)	10 (6.6–13.4)	11.8 (8.9–14.6)	0.37
TNF-α (pg/mg protein)	7.5 (5.4–9.5)	5 (3.5–6.5)	5.8 (3.8–7.8)	6 (4.2–7.8)	6.2 (4.9–7.6)	0.30
4-HNE/GAPDH	0.47 [0.36–0.59]	0.57 [0.44–0.69]	0.52 [0.41–0.62]	0.47 [0.39–0.55]	0.53 [0.41–0.64]	0.38
GPX (U/mg protein)	1.4 (1–1.9)	1.6 (1.1–2.1)	1.5 (1–1.9)	1.3 (0.6–1.9)	1.5 (1–2)	0.88
CAT (nmol/min/mg protein)	190 (158–222)	215 (157–274)	193 (156–230)	183 (136–229)	194 (147–241)	0.83
SOD (U/mg protein)	38.9 [34.3–43.5]	43.4 [36.7–50.2]	40.2 [33.1–47.2]	44.7 [34–55.5]	35.3 [28.3–42.3]	0.34
VEGF (pg/mg protein)	3.6 (1.6–5.6)	2.5 (1.2–3.8)	2.1 (0.9–3.3)	2.2 (0.7–3.7)	3.7 (1.7–5.7)	0.31

4-HNE: 4-hydroxynonenal; CAT: catalase; GPX: glutathione peroxidase; IL-6: interleukin 6; IL-10: interleukin 10; IL-1β: interleukin 1 beta; SOD: superoxide dismutase; TNF-α: tumor necrosis factor alpha; VEGF: vascular endothelial growth factor. *p*: *p*-value ANOVA. The data between parentheses and brackets represent the 95% confidence intervals for the mean and trimmed mean, respectively.

## Data Availability

The data that support the findings of this study are available from the corresponding author upon reasonable request.

## Supplementary Materials

The following supporting information can be downloaded at: https://www.mdpi.com/article/10.3390/biomedicines13092221/s1, Figure S1: Mortality up to seven days after myocardial infarction surgery. (A1) Mortality stratified by group. (A2) Proportion of animals in each group stratified by outcome, with animals that survived (left column) and those that died due to myocardial infarction (right column); Figure S2: Body mass. (A) Body mass of the rats throughout the study weeks (mean with standard deviation). (B) Body mass after training period (Week9). (C) Body mass 6 days after myocardial infarction (Week10); Figure S3: (A) Representative recordings of left ventricular and aortic pressures; (B) Heart rate (HR) in hemodynamic assessment; Figure S4: Representative recordings of (A) LAESA, left atrial area at end-systole; (B) LVEDA, left ventricular end-diastolic area at the papillary muscle level; (C) LVESA, left ventricular end-systolic area at the papillary muscle level; Table S1: Sample size for each group; Table S2: Blinded procedures.
