# Supplementary material for: Prior Aerobic Exercise Training Fails to Confer Cardioprotection Under Varying Exercise Volumes in Early Post-Infarction Cardiac Remodeling in Female Rats
Source: Biomedicines. 2025 Sep 10;13(9):2221. doi: 10.3390/biomedicines13092221 (PMC12467140; doi:10.3390/biomedicines13092221)
Supplement: Supplementary file 1 [file biomedicines-13-02221-s001.zip › biomedicines-3790642-supplementary/Supplemental Figures S1–S4.pdf]

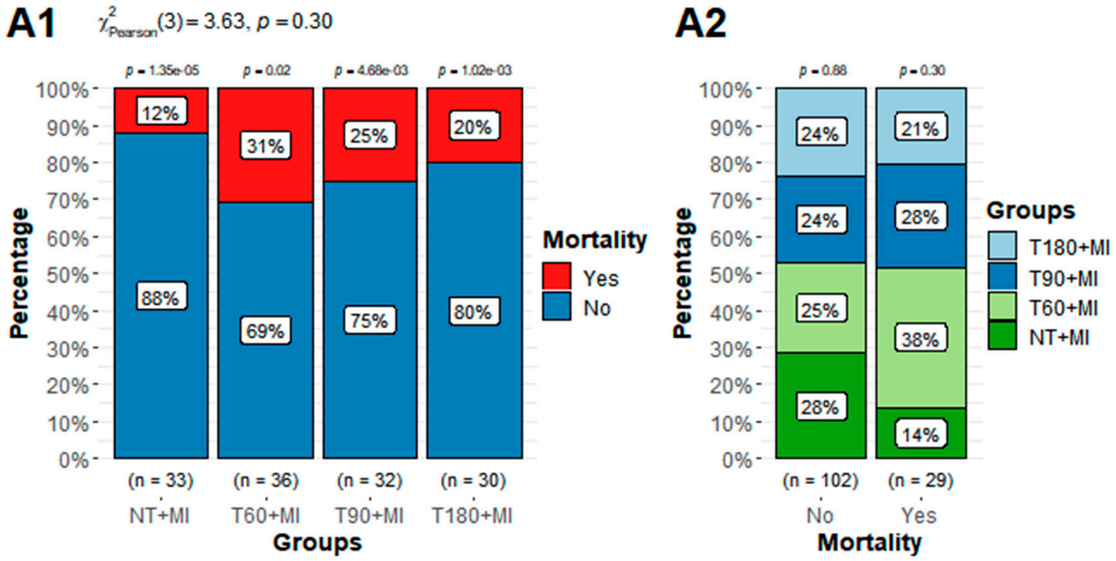

Figure S1. Mortality up to seven days after myocardial infarction surgery. (**A1**) Mortality stratified by group. (**A2**) Proportion of animals in each group stratified by outcome, with animals that survived (left column) and those that died due to myocardial infarction (right column).

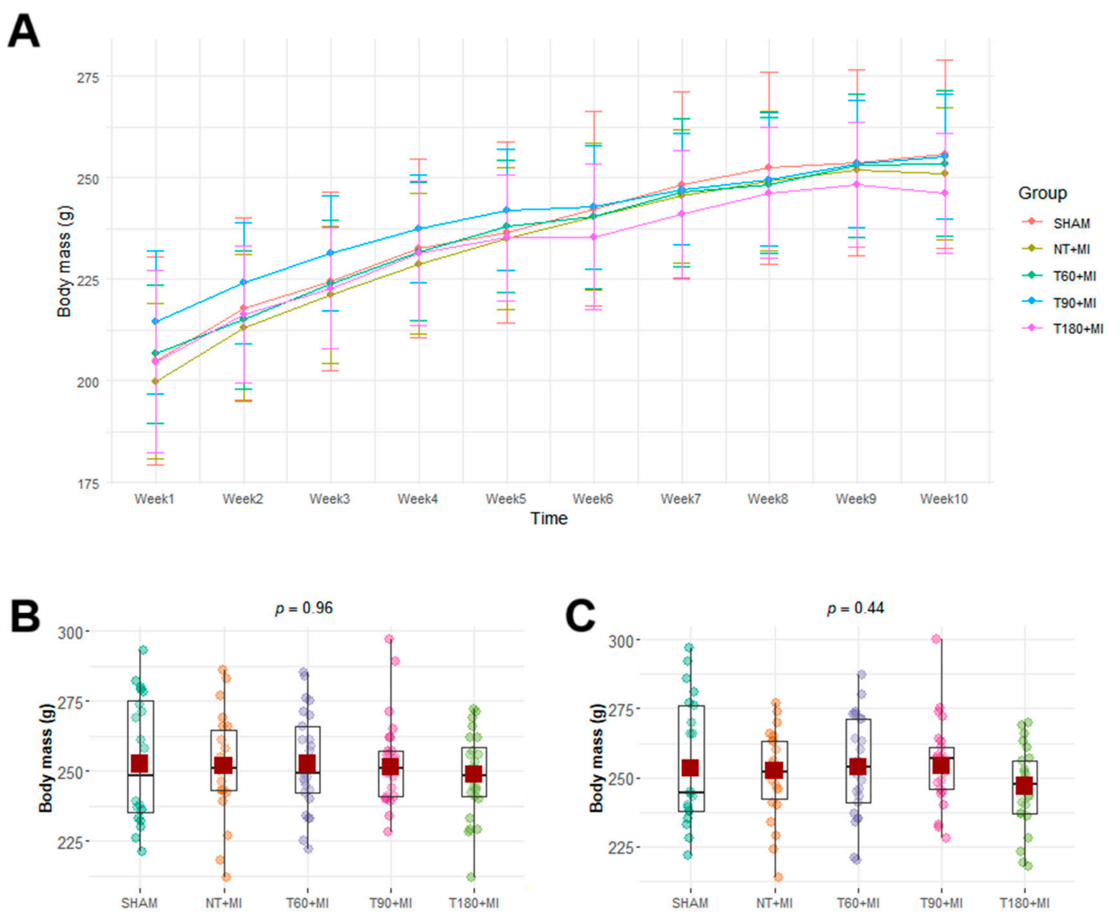

Figure S2. Body mass. (**A**) Body mass of the rats throughout the study weeks (mean with standard deviation). Week1: first week of training; Week9: week of myocardial infarction induction surgery; Week10: week of euthanasia. (**B**) Body mass after training period (Week9). (**C**) Body mass 6 days after myocardial infarction (Week10). Boxplots with individual data points and 20% trimmed means (red squares). The *p*-value displayed above the boxplots refers to the ANOVA. **B** and **C** charts: Welch's ANOVA with trimmed means and pairwise comparisons via Yuen's trimmed test.

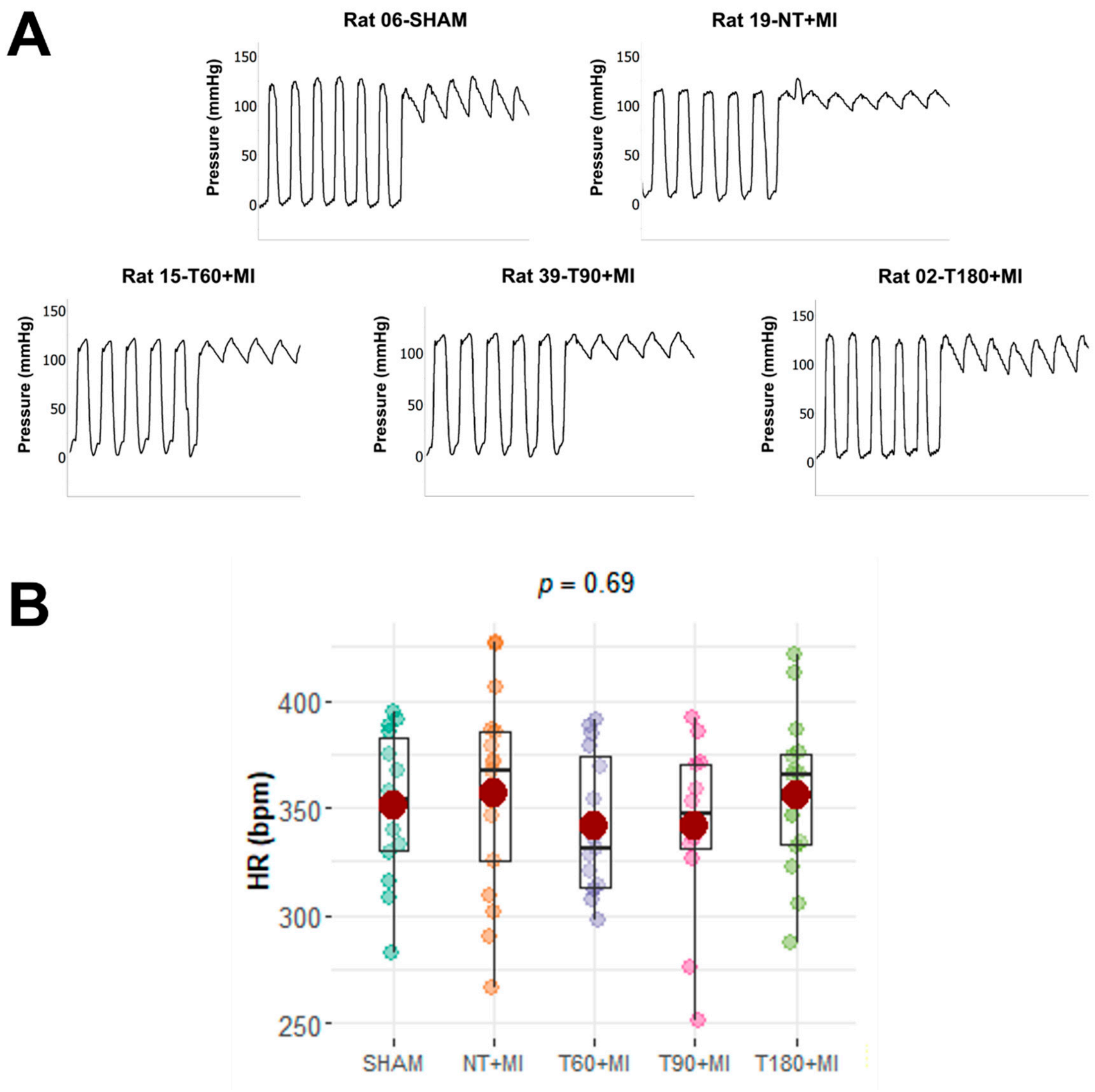

Figure S3. (A) Representative recordings of left ventricular and aortic pressures; (B) Heart rate (HR) in hemodynamic assessment. Boxplots with individual data points and means (red circles). The *p*-value displayed above the boxplots refers to the ANOVA. Fisher's one-way ANOVA and pairwise comparisons via Student's t test.

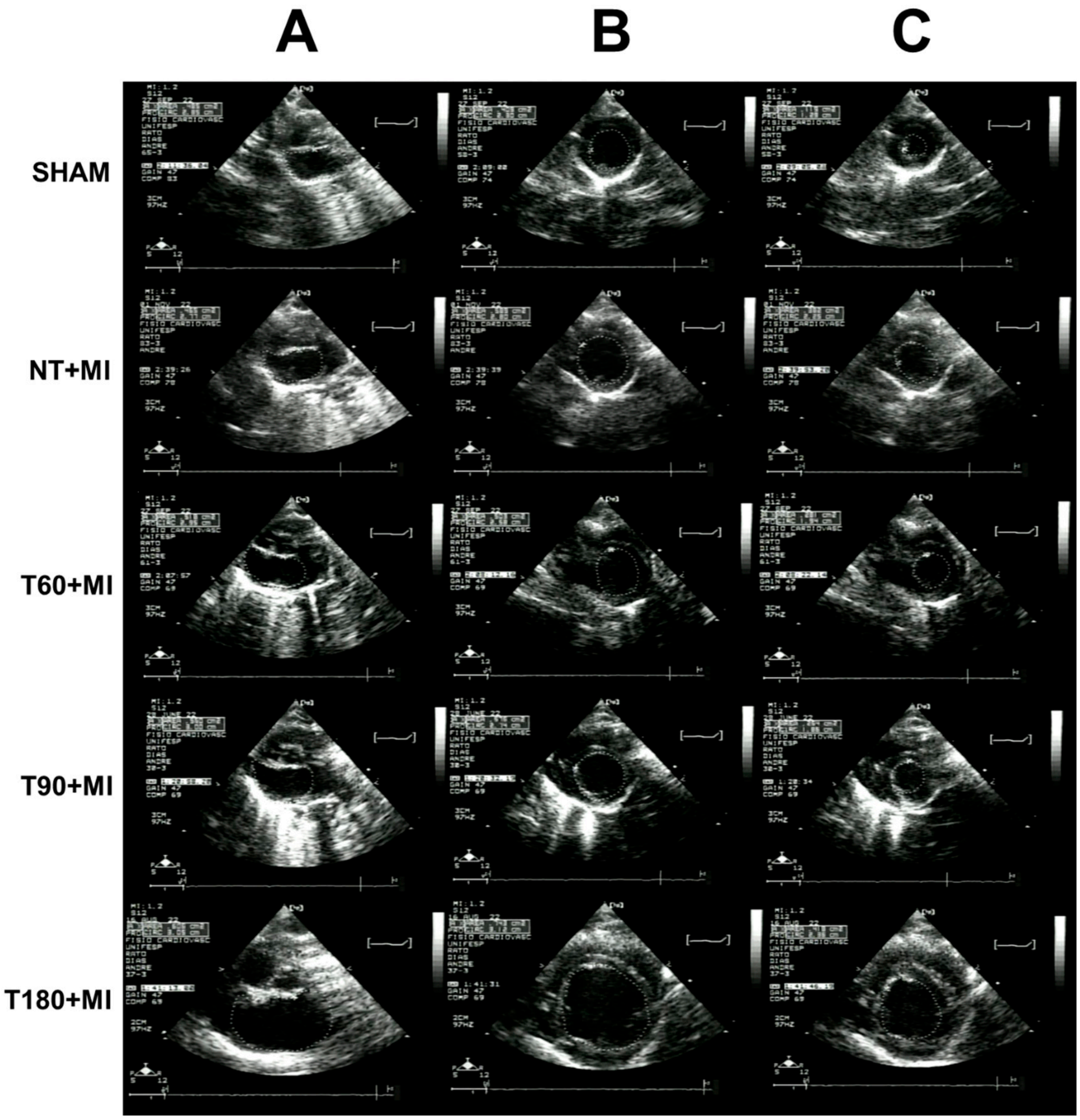

Figure S4. Representative recordings of (A) LAESA, left atrial area at end-systole; (B) LVEDA, left ventricular end-diastolic area at the papillary muscle level; (C) LVESA, left ventricular end-systolic area at the papillary muscle level.
