# Supplementary material for: Prior Aerobic Exercise Training Fails to Confer Cardioprotection Under Varying Exercise Volumes in Early Post-Infarction Cardiac Remodeling in Female Rats
Source: Biomedicines. 2025 Sep 10;13(9):2221. doi: 10.3390/biomedicines13092221 (PMC12467140; doi:10.3390/biomedicines13092221)
Supplement: Supplementary file 1 [file biomedicines-13-02221-s001.zip › biomedicines-3790642-supplementary/Supplemental Tables S1–S2.pdf]

Table S1. Sample size for each group.

	SHAM	NT+MI	T60+MI	T90+MI	T180+MI
**Mortality**	-	33	36	32	30
**Body mass**	20	22	25	25	24
**SMM**	14	16	14	12	13
**CPET time**	28	33	35	33	30
**VO_2peak**	24	28	25	17	17
**LVM**	15	15	13	12	13
**RVM**	15	15	13	12	13
**LWC**	15	16	14	12	13
**E-wave**	10	13	10	7	12
**A-wave**	9	12	10	6	12
**E/A**	9	12	10	6	12
**DT**	10	13	10	7	12
**IVRT**	10	13	10	7	12
**MI size**	-	13	10	7	12
**LAESA**	10	13	10	7	12
**LVEDA**	10	13	10	7	12
**LVESA**	10	13	10	7	12
**FAC**	10	13	10	7	12
**LVSP**	14	17	15	12	15
**LVEDP**	14	17	15	12	15
**+dP/dt**	14	17	15	12	15
**-dP/dt**	14	17	15	12	15
**SBP**	14	17	15	12	15
**DBP**	14	17	15	12	15
**HR**	14	17	15	12	15
**IL-10**	13	14	15	10	14
**IL-6**	13	14	15	10	14
**IL-1β**	13	14	15	10	14
**TNF-α**	13	14	15	10	14
**GPX**	13	15	14	10	14
**CAT**	10	10	10	10	10
**SOD**	10	10	10	10	10
**VEGF**	8	9	8	7	7
**4-HNE/GAPDH**	8	8	7	8	9
**PLB/GAPDH**	8	8	7	8	9
**PLB(THR17/SER16)/GAPDH**	8	8	7	8	9
**NCX1/GAPDH**	8	8	7	8	9
**SERCA2a/GAPDH**	8	8	7	8	9
**LTCC/GAPDH**	8	8	7	8	9
**RyR/GAPDH**	8	8	7	8	9

Table S2. Blinded procedures.

Procedure	Blinded	Responsible researcher
Cardiopulmonary exercise test	No	First author
Myocardial infarction surgery	Yes	Second author
Ecocardiography	Yes	Fifth author
Left ventricular hemodynamics	Yes	Second author
Euthanasia and collection of biological materials	Yes	Second author
Soleus muscle mass and body mass measurements	No	First author
ELISA	Yes	Fourth author
Antioxidant enzyme activity	Yes	Fourth author
Western blotting revelation	Yes	Third author
Western blotting image quantification	No	First author
