# Supplementary material for: Prior Aerobic Exercise Training Fails to Confer Cardioprotection Under Varying Exercise Volumes in Early Post-Infarction Cardiac Remodeling in Female Rats
Source: Biomedicines. 2025 Sep 10;13(9):2221. doi: 10.3390/biomedicines13092221 (PMC12467140; doi:10.3390/biomedicines13092221)

WESTERN BLOTS IMAGES

Red rectangles were added to the images to highlight the representative western blot samples used in Figure 5.

A green arrow ( ) was used to indicate the analyzed band, except for 4-HNE, where the intensity of all bands marked by 4-HNE was quantified (global oxidative stress).

Molecular weight markers (Bio-Rad, catalog #1610375)

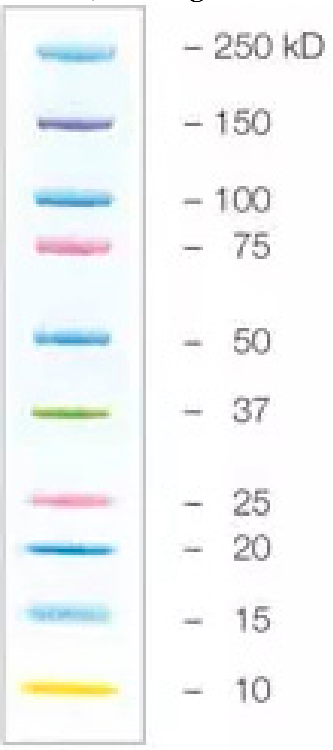

4-HNE

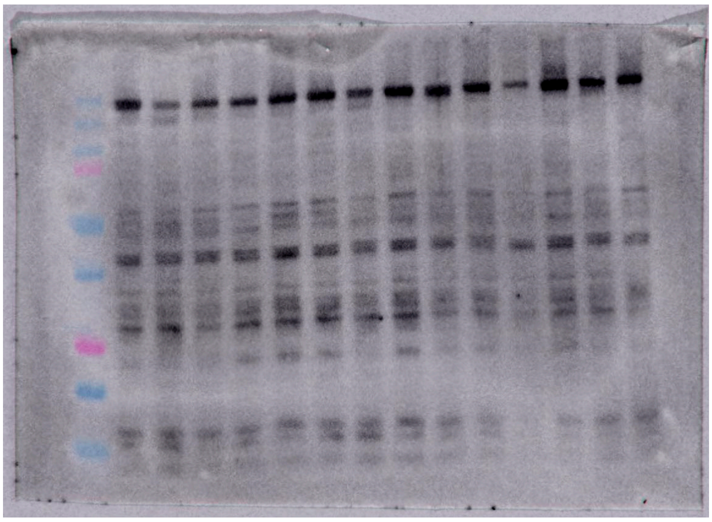

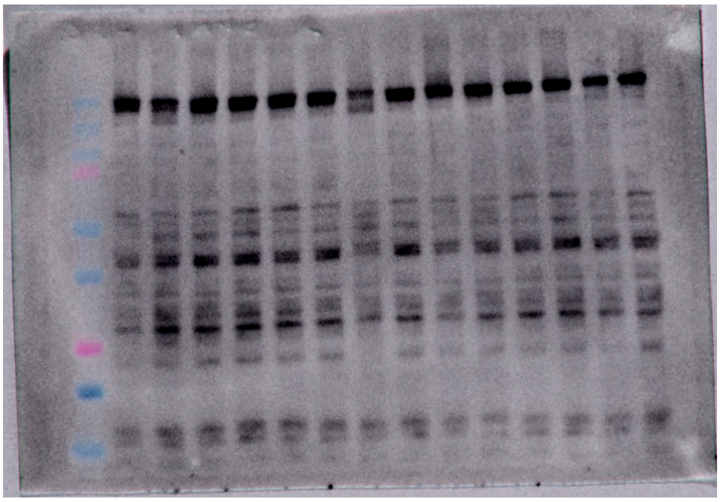

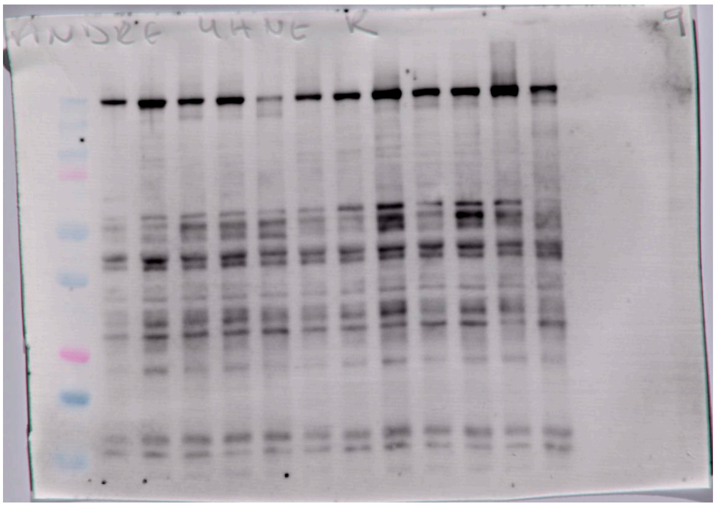

GAPDH (4-HNE)

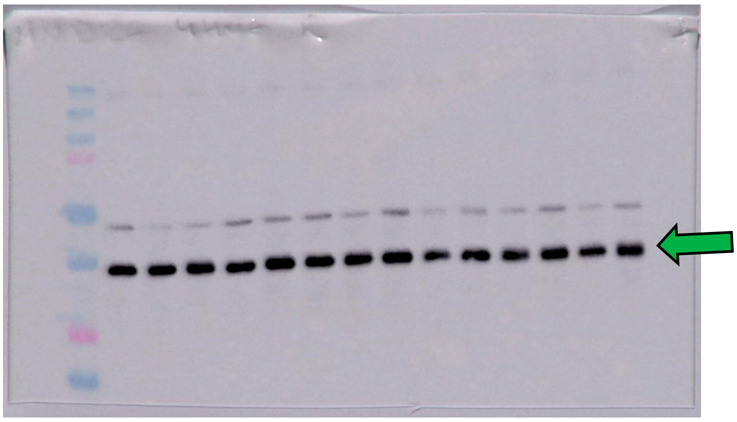

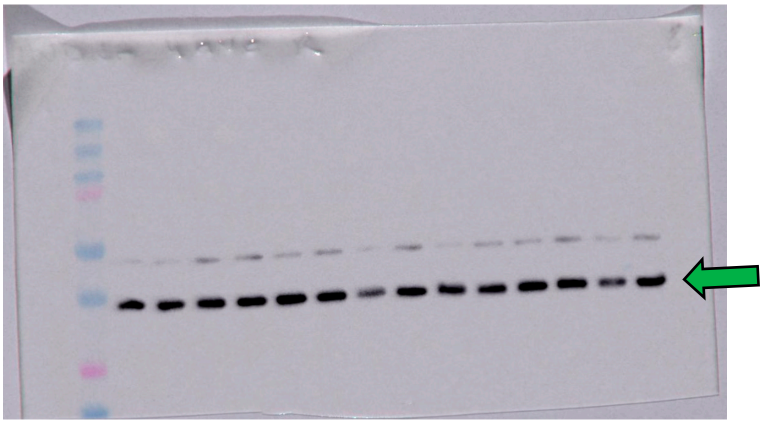

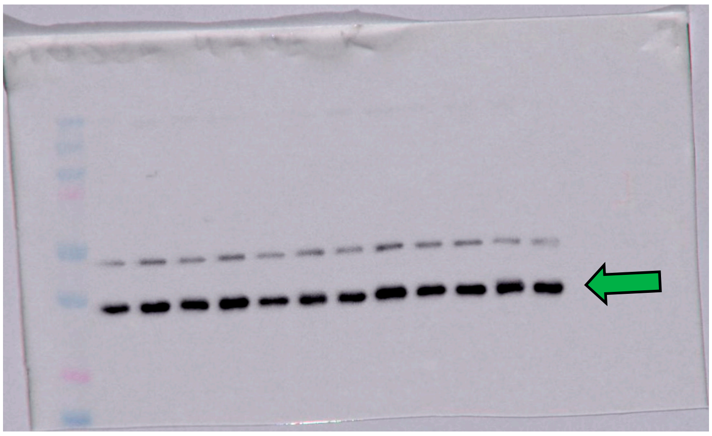

LTCC

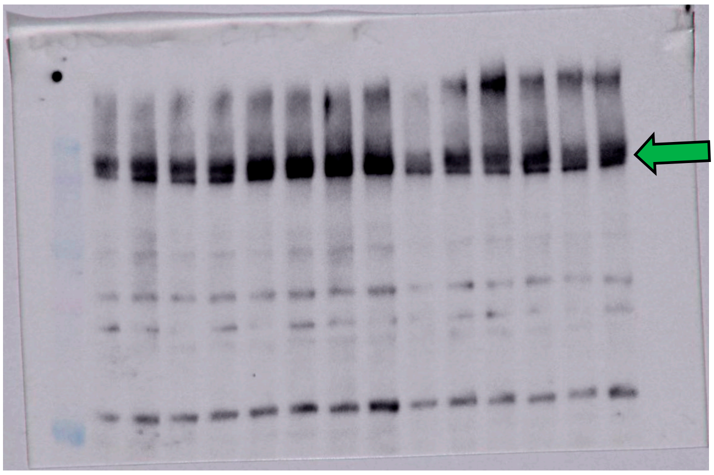

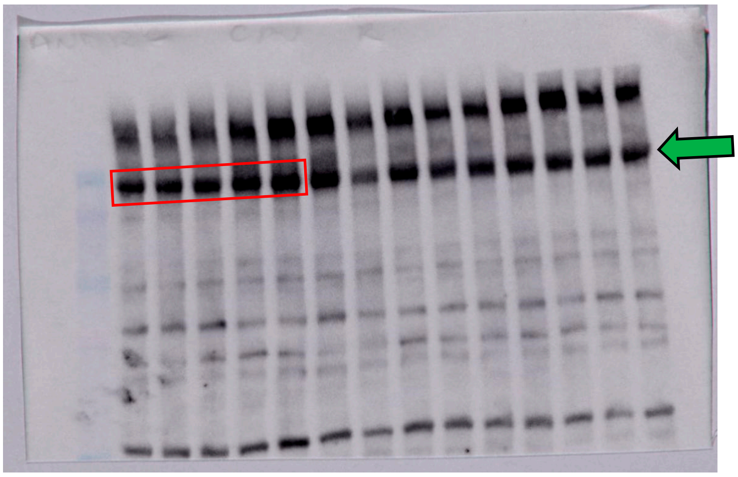

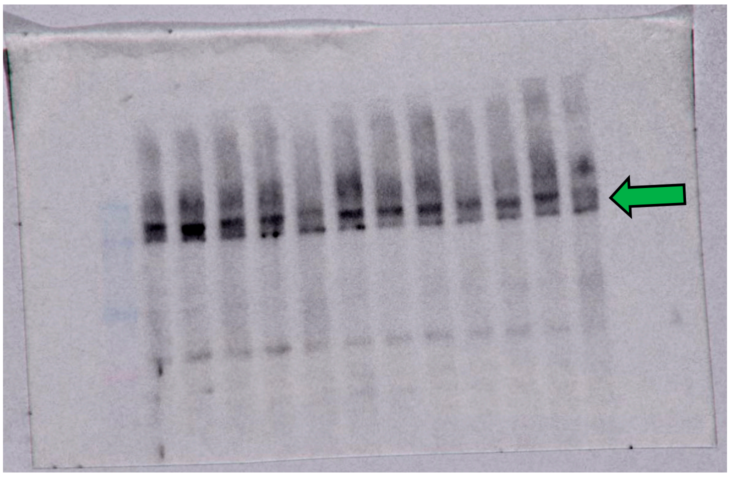

GAPDH (LTCC , RyR)

The same membrane was used for GAPDH-LTCC and GAPDH-RyR.

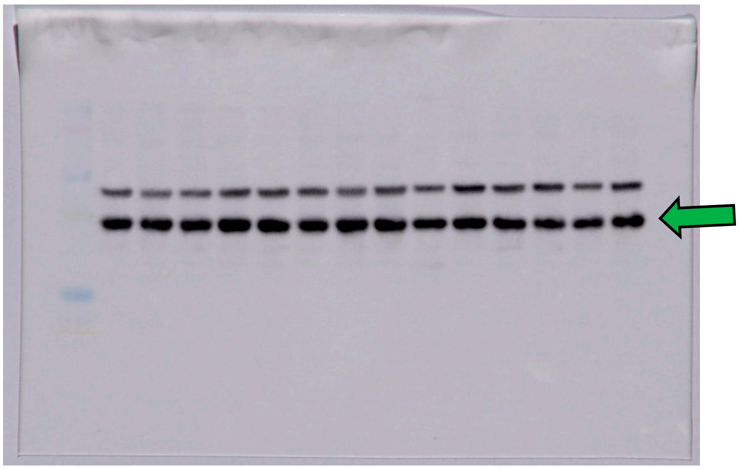

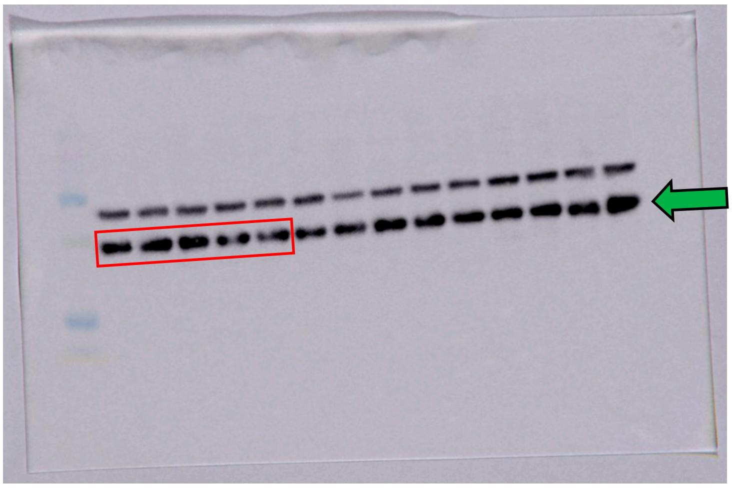

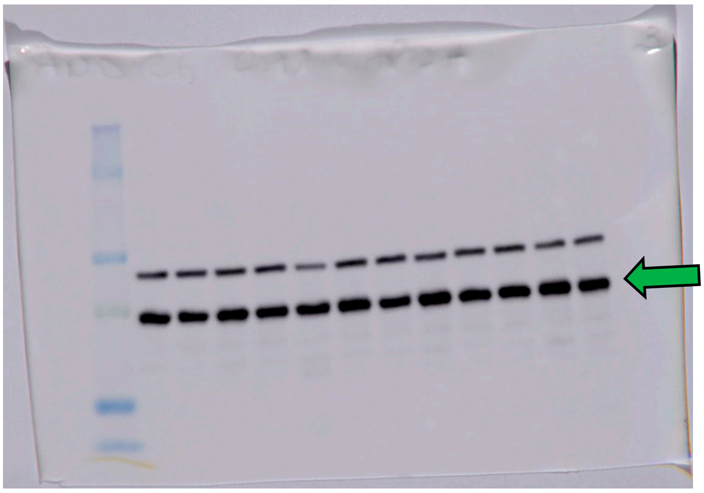

NCX1

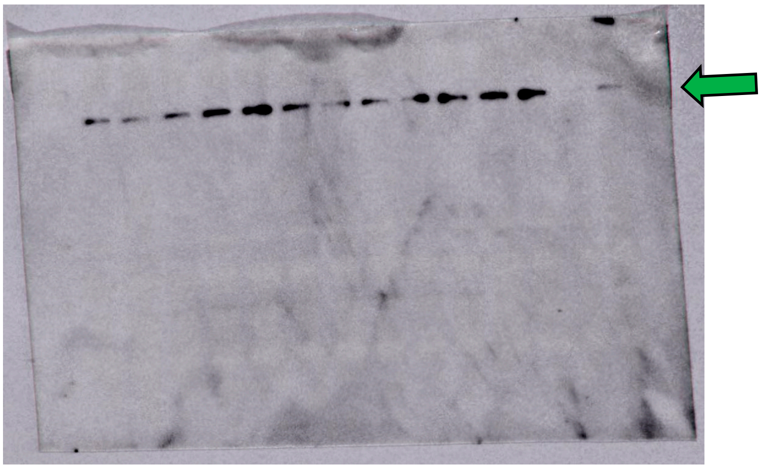

Western Blot Stripping

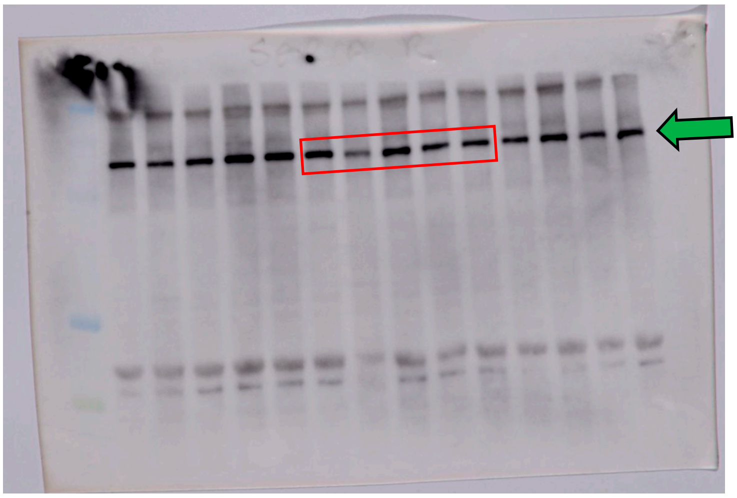

Western Blot Stripping

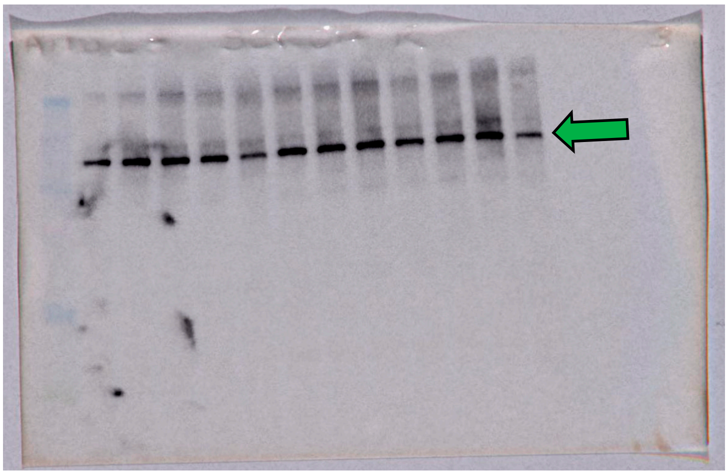

GAPDH (NCX1)

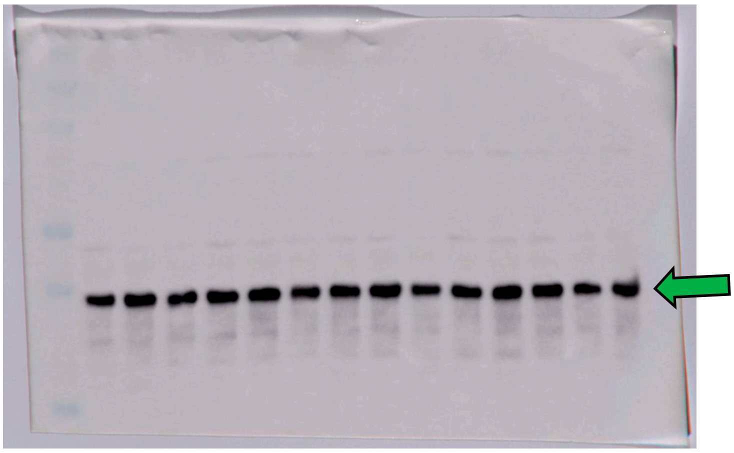

The same membrane was used for GAPDH-NCX1 and GAPDH-SERCA2a.

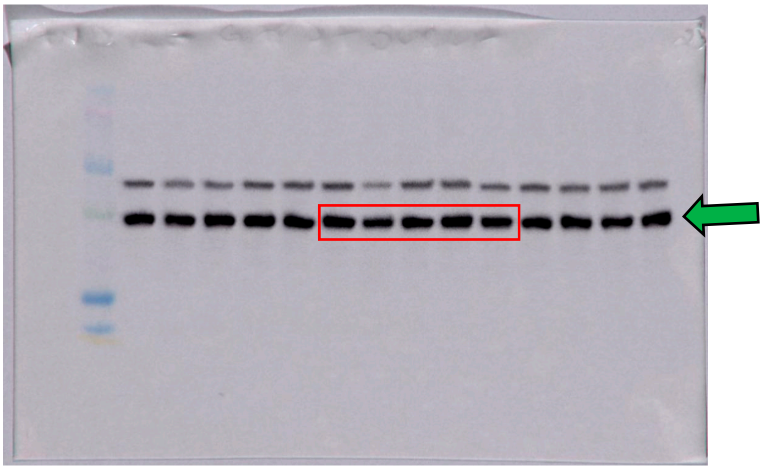

The same membrane was used for GAPDH-NCX1 and GAPDH-SERCA2a.

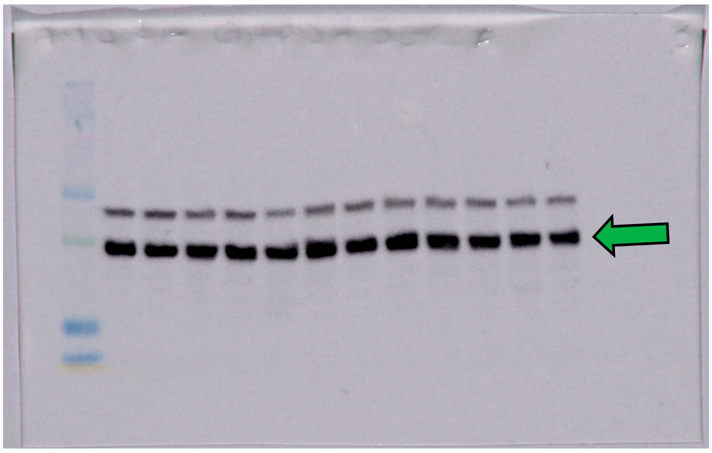

PLB(Thr17/Ser16)

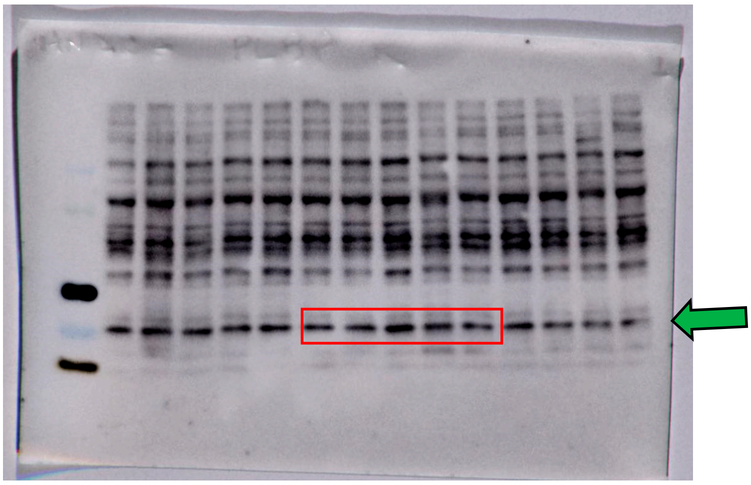

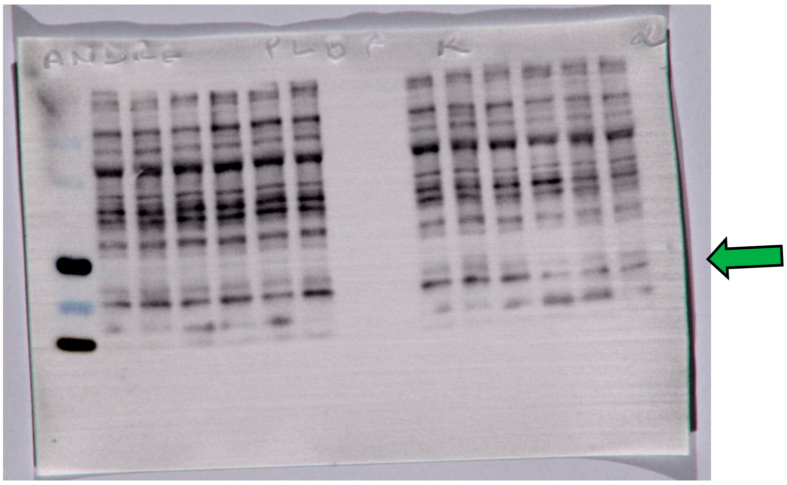

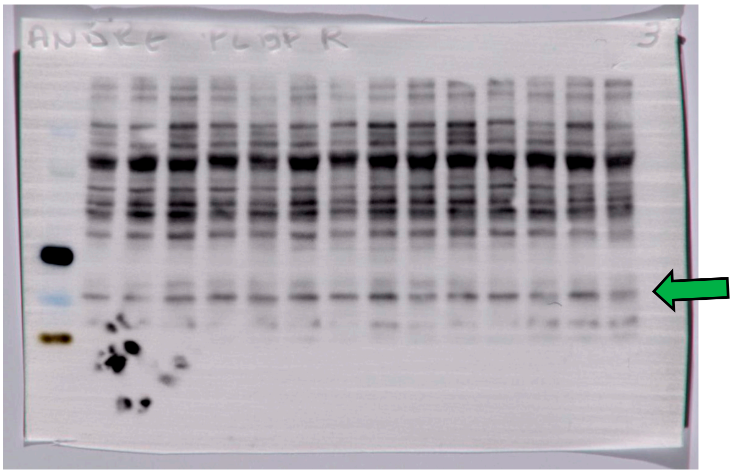

GAPDH (PLB(Thr17/Ser16)

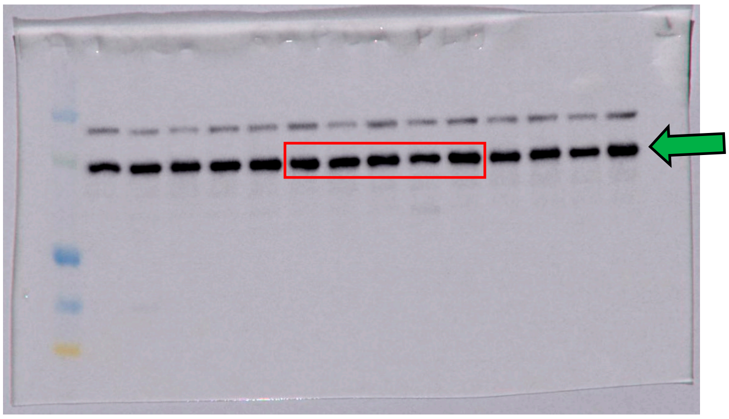

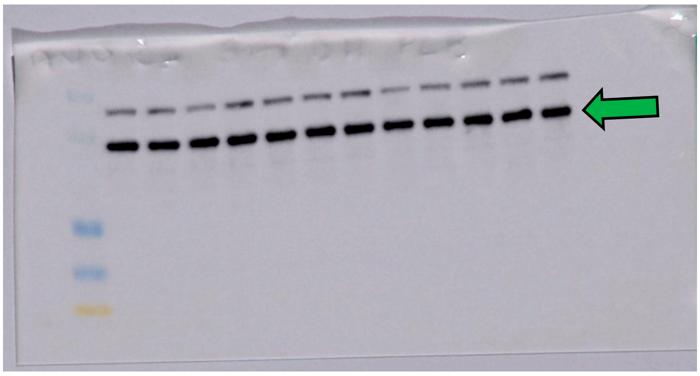

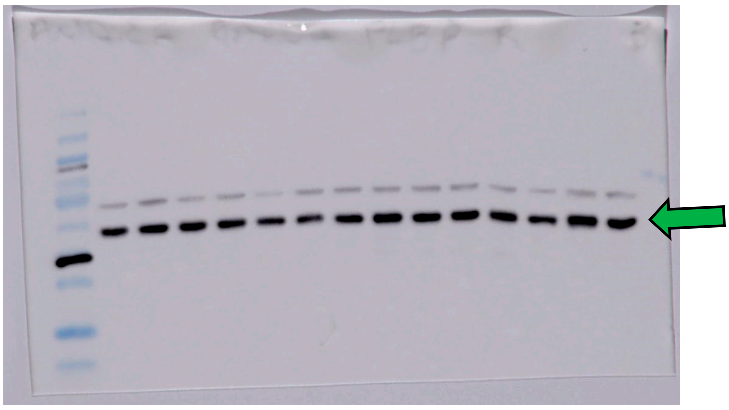

PLB

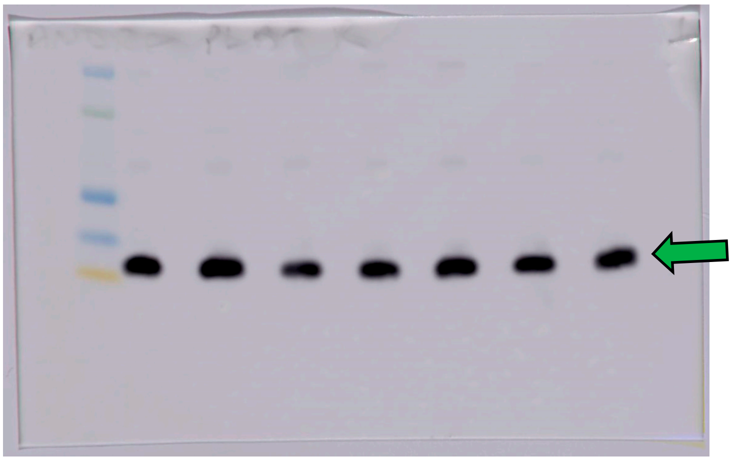

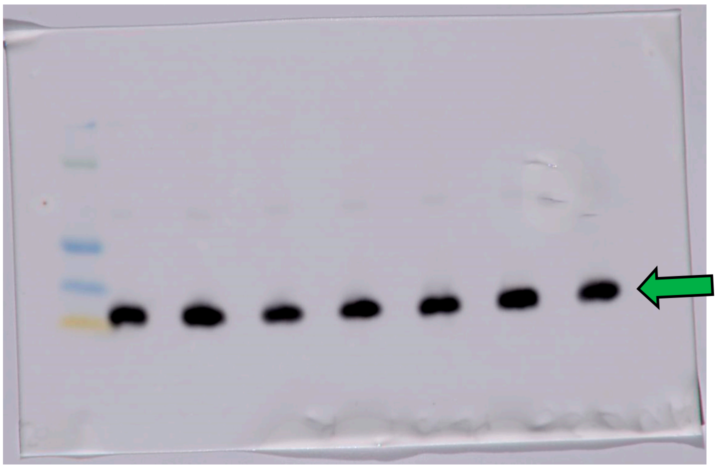

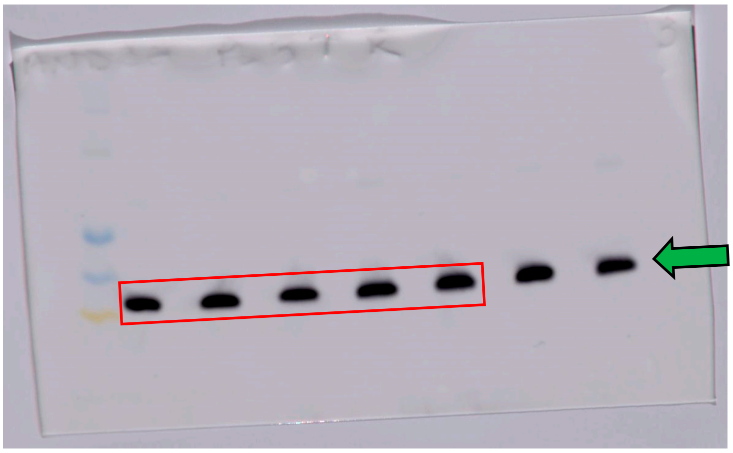

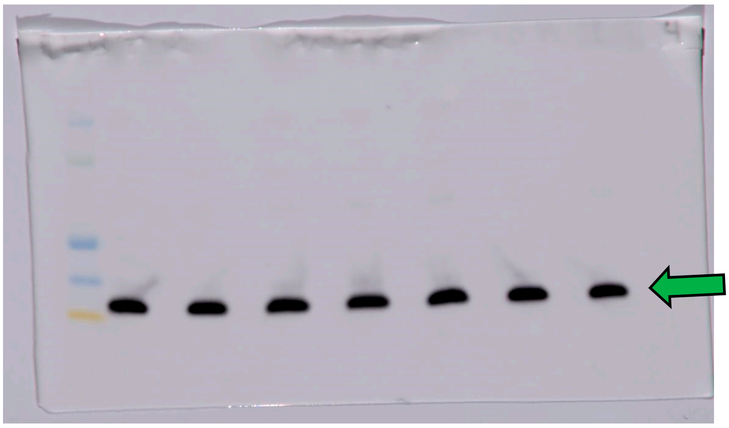

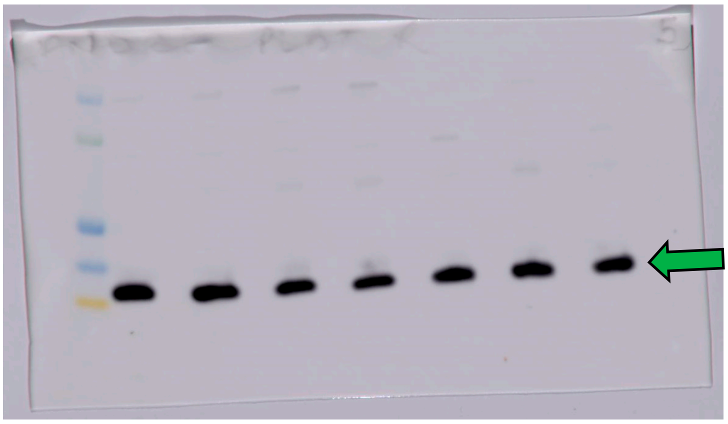

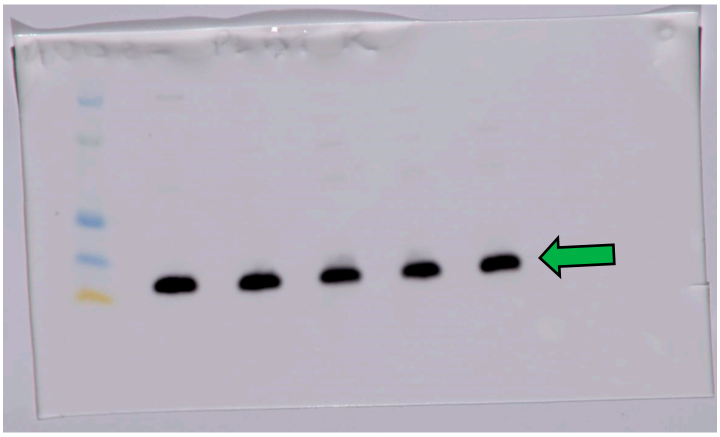

GAPDH (PLB)

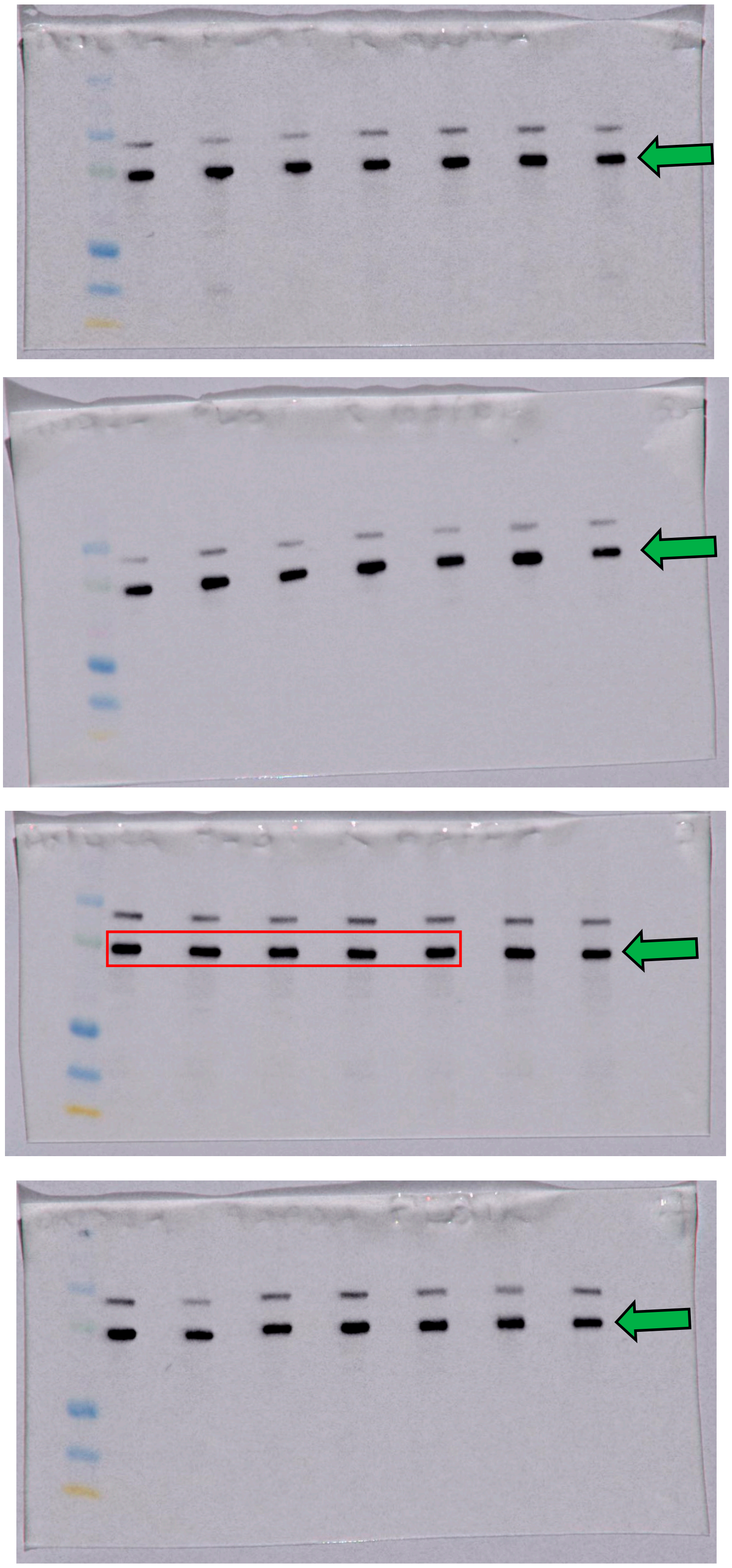

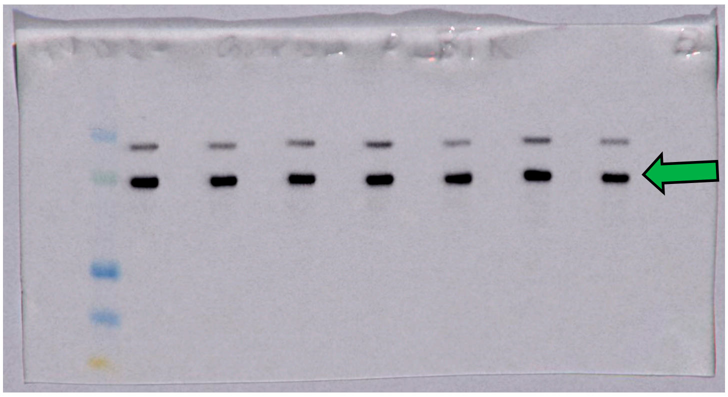

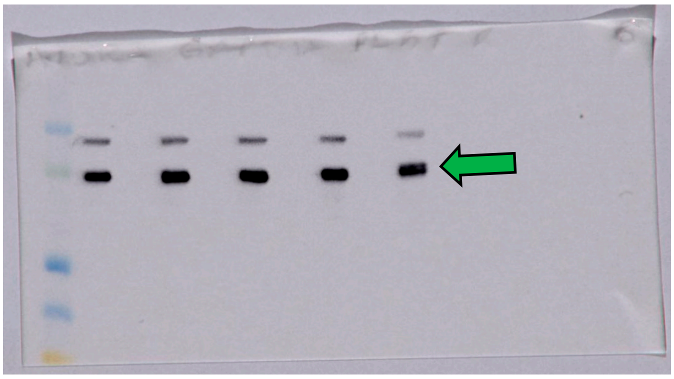

RyR (Western Blot Stripping)

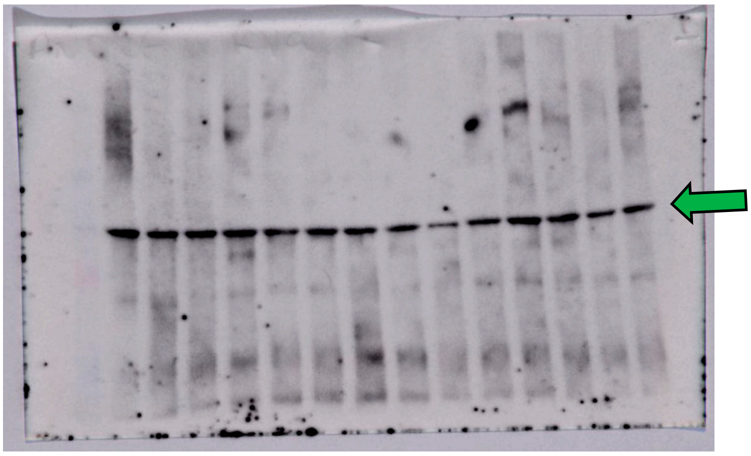

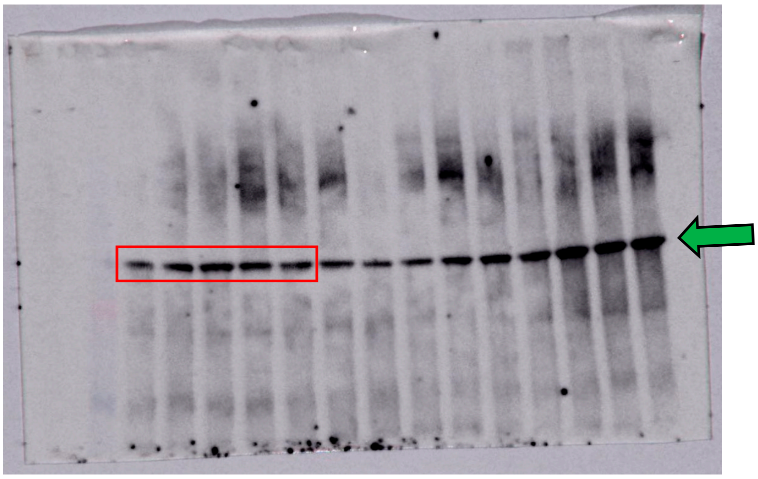

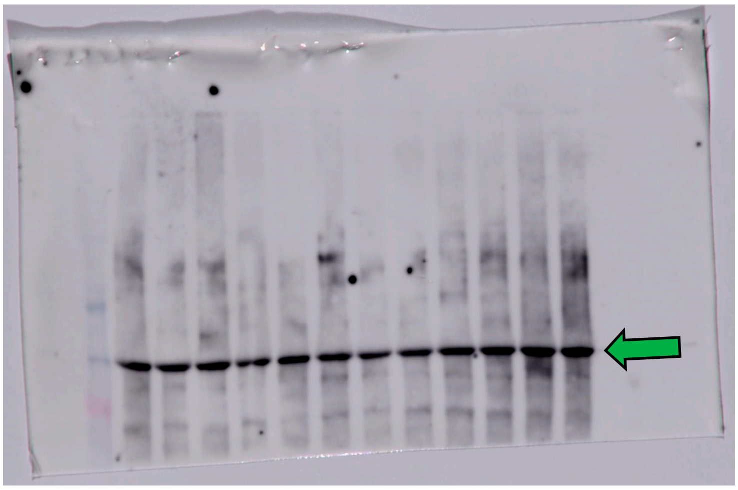

SERCA2a

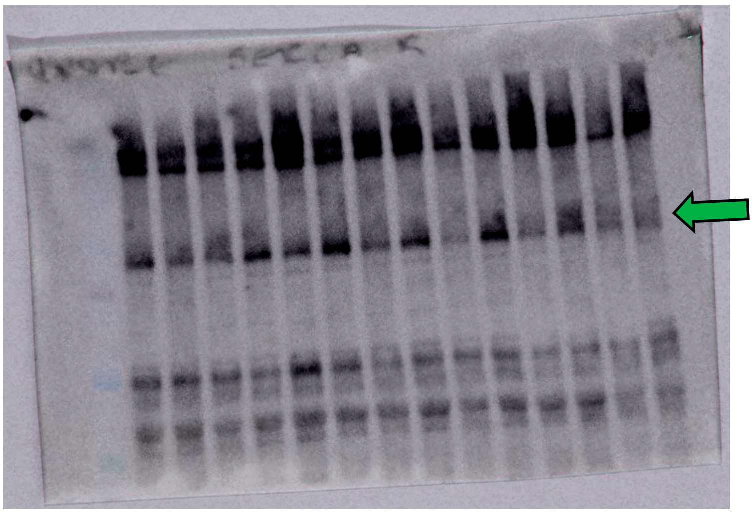

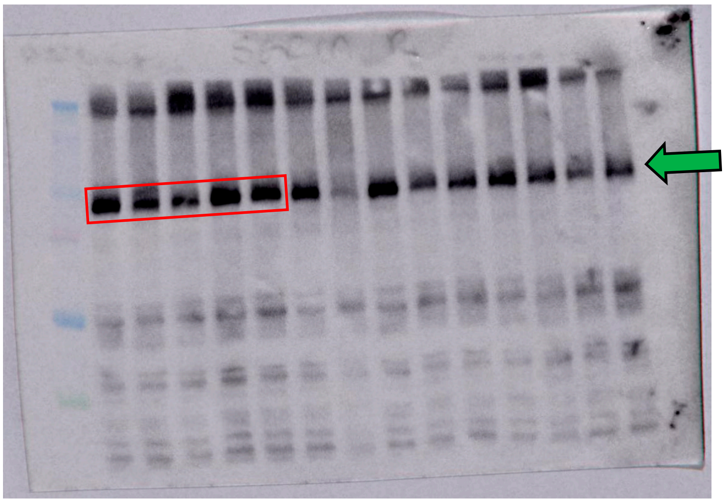

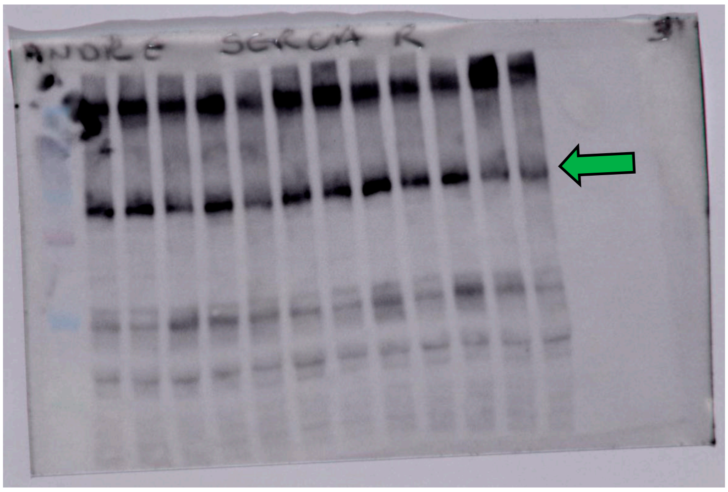

GAPDH (SERCA2a)

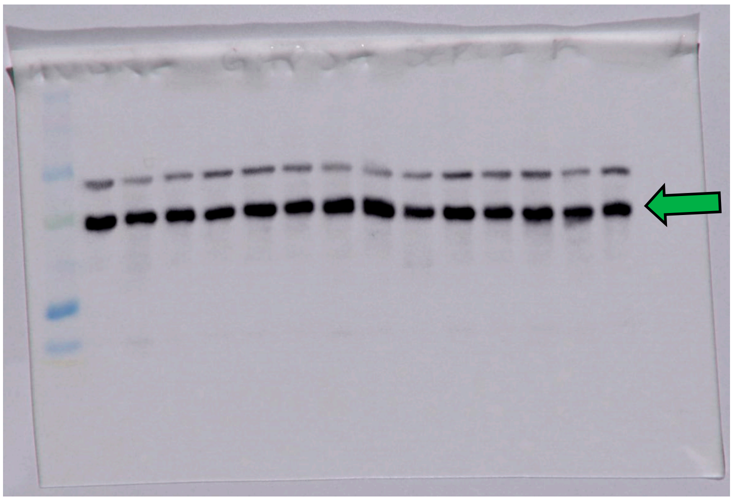

The same membrane was used for GAPDH-NCX1 and GAPDH-SERCA2a.

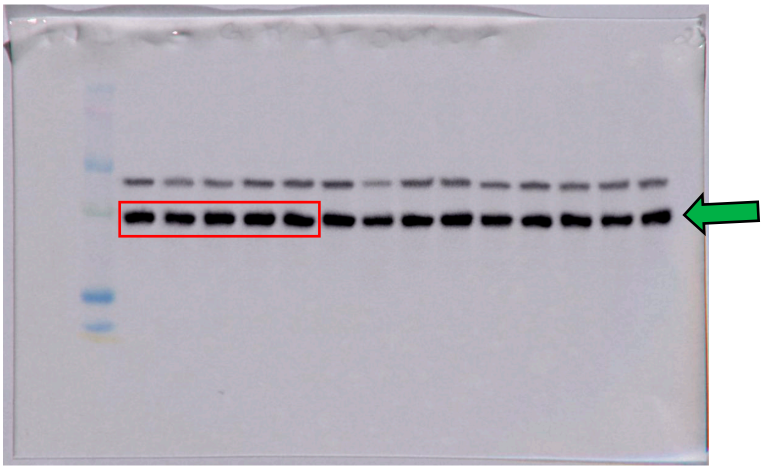

The same membrane was used for GAPDH-NCX1 and GAPDH-SERCA2a.

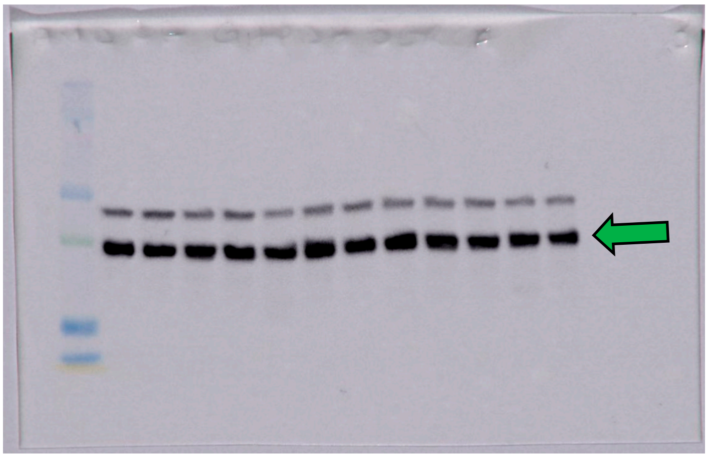

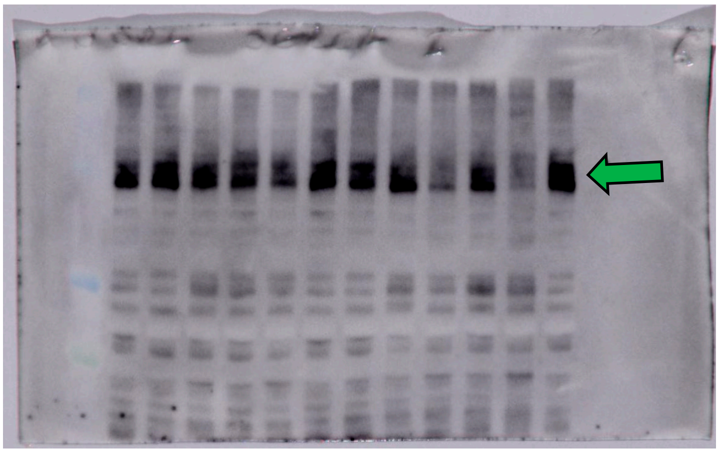

Supplement: Supplementary file 1 [file biomedicines-13-02221-s001.zip › biomedicines-3790642-supplementary/western_blot_images.pdf]
